# Comparing Bayesian estimation and structural-after-measurement approaches for structural equation models with latent interactions and complex data structures

**DOI:** 10.3758/s13428-025-02840-x

**Published:** 2025-10-22

**Authors:** Kyle Cox, Benjamin Kelcey

**Affiliations:** 1 University of North Carolina at Charlotte, Charlotte, NC USA; 2 University of Cincinnati, Cincinnati, OH USA

**Keywords:** Structural-after-measurement (SAM) approaches, Bayesian estimation, Latent interaction, Multilevel structural equation model, Partially nested data

## Abstract

Bayesian and structural-after-measurement (SAM) approaches have been developed, in part, to address limitations of conventional estimators in the context of structural equation models (SEMs) with latent interactions. Although both approaches have shown promise in a variety of contexts including small-sample studies, there is very little literature systematically comparing the relative benefits, limitations, and trade-offs among these approaches. In this study, we (a) compared the performance of estimators under each approach in multilevel SEMs with a within-, between-, or cross-level latent interaction and (b) demonstrated the flexibility of SAM approaches by extending and investigating them in partially nested SEMs with latent moderated mediation. The results suggest substantial differences between estimator performance as a function of the type of latent interaction. SAM approaches performed well with a variety of latent interactions in multilevel and partially nested SEMs, while Bayesian approaches, including those with informative priors, struggled with models that included a cross-level latent interaction and were not easily extended to partially nested SEMs. Overall, the results suggest that SAM approaches are a versatile and highly adaptable alternative or complement to conventional full-information estimators. To conclude, we outline estimator considerations based on the SEM type, latent interaction, and data structure.

**Supplementary Information:**

The online version contains supplementary material available at 10.3758/s13428-025-02840-x.

Advancements in estimation strategies for structural equation models have sought to address the difficulty of consistently estimating model parameters with latent interactions. Prior research with maximum likelihood (ML) estimation, for example, has demonstrated a broad range of computational challenges (e.g., high-dimensional integration, non-convergence, impermissible parameter values) and instability and/or bias in parameter estimates with models using latent interactions (Asparouhov & Muthén, 2020; Cox & Kelcey, 2021; Cox et al., 2023; Rosseel et al., 2025). Further, literature has widely demonstrated that estimation issues tend to be exacerbated in sample sizes that are small (e.g., fewer than 100 clusters) relative to the overall model complexity (e.g., Bogaert et al., 2022; Cox & Kelcey, 2021; Cox et al., 2023; Devlieger & Rosseel, 2017, 2020; Rosseel, 2020; Rosseel et al., 2025; Smid & Rosseel, 2020).

The core issue is that full-information estimators typically rely on large sample theory (e.g., requiring at least several hundred clusters or strong priors) while most behavioral studies draw on what is considered to be statistically “small” sample sizes (e.g., less than 100 clusters, with 50 individuals per cluster). Prior literature has extensively documented the limitations of extant estimators with small datasets and concluded that this mismatch has the potential to produce erroneous inferences and ultimately impede scientific discovery, innovation, and reproducibility (e.g., Aguinis et al., 2017; Rosseel, 2020; Smid & Rosseel, 2020; van de Schoot & Miocevic, 2020).

Estimation issues intensify when more complex data structures are present (e.g., multilevel or partial nesting; Devlieger & Rosseel, 2020; Kelcey et al., 2021). Prior research has widely suggested that estimation in multilevel structural equation models (MLSEMs) with samples of less than 100 clusters is often untenable (e.g., Kline, 2011). However, literature from a broad range of disciplines has emphasized and demonstrated that small-scale studies offer critical contributions to theory, program development, and social issues when well executed (e.g., Bodner & Bliese, 2018; van de Schoot & Miocevic, 2020). In many areas of research, samples of less than 100 clusters (e.g., therapists, teachers, counselors) predominate, and samples greater than 100 clusters are often prohibitively expensive or impractical for most studies (e.g., Schochet, 2011).

Two alternative approaches to ML for estimating SEMs including those with latent interactions are (a) structural-after-measurement (SAM) approaches (Rosseel & Loh, 2024; Rosseel et al., 2025) and (b) Bayesian estimation (Asparouhov & Muthén, 2020). Specifically, recent work has extended a local SAM estimator that utilizes factor score regression with a Croon-based correction (Croon, 2002) to a diverse range of (multilevel) SEMs (e.g., Devlieger & Rosseel, 2017; Kelcey, 2019, Kelcey et al., 2021) including those with latent interactions (Cox & Kelcey, 2021; Cox et al., 2023; Rosseel et al., 2025). The SAM-Croon estimator has outperformed other common estimators (e.g., ML) in multiple settings (e.g., Cox & Kelcey, 2021; Cox et al., 2023; Devlieger et al., 2016; Devlieger & Rosseel, 2017, 2020; Kelcey, 2019; Kelcey et al., 2021; Loncke et al., 2018; Lu et al., 2011; Takane & Hwang, 2018).

Bayesian approaches have also been developed for SEMs that include latent interactions (Asparouhov & Muthén, 2020; Cox & Kelcey, 2021; Smid & Rosseel, 2020). However, few studies have probed the performance of Bayesian estimation with limited sample sizes and more complex MLSEMs with latent interactions (e.g., multiple latent variables with multiple indicators). Prior literature on Bayesian approaches has highlighted the importance of accurate and informative priors especially with limited sample sizes and noted issues when using Bayesian estimation with naïve priors (e.g., Smid et al., 2020; Smid & Winter, 2020). Nevertheless, Bayesian approaches have been successfully implemented for a variety of MLSEMs including those with different types of latent interactions (Asparouhov & Muthén, 2020).

Although there have been some limited comparisons between SAM-based and Bayesian methods in simple settings (e.g., Smid & Rosseel, 2020), largely absent from the literature is a direct comparison of SAM and Bayesian approaches for SEMs with latent interactions under increasingly complex but authentic data structures (e.g., multilevel or partial nesting). A direct comparison of SAM and Bayesian estimators for MLSEMs with latent interactions would better map out conditions under which each approach is advantageous, detrimental or similar, and general advantages and disadvantages of both approaches. In addition, conventional applications and developments of MLSEM have historically focused on simple (fully) nested two- or three-level structures such as team members within a team (within an organization) or patients nested within a therapist. Literature has noted the prevalence of more complex structures such as partially nested, cross-classified, and many-level SEMs (e.g., Mehta & Petscher, 2016; Petscher & Schatschneider, 2019; Pritikin et al., 2017). Estimators that can be easily adapted to various data structures such as partial nesting provide a valuable tool for researchers.

Given these gaps, the purpose of this study is to (a) compare Bayesian and SAM approaches for MLSEMs that include latent interactions and (b) extend SAM approaches to more complex models (i.e., latent moderated mediation) in partially nested SEMs. As part of our extension of SAM approaches, we highlight the limitations of Bayesian estimation in these contexts. The paper is organized into two main sections reflecting its purpose. The first section introduces SAM approaches and then utilizes a series of three Monte Carlo simulation studies to examine SAM and Bayesian estimator performance with MLSEMs that include a (1) between-level, (2) within-level, or (3) cross-level latent interaction. The second section describes the extension of SAM approaches for partially nested SEMs with a latent moderated mediation model and includes an additional Monte Carlo simulation study to probe the performance of the SAM approach in this context. We conclude with general recommendations and considerations for estimating structural equation models with latent interactions and complex data structures.

## Multilevel structural equation models with latent interactions

To compare Bayesian and SAM approaches for MLSEMs that include latent interactions, we considered two-level SEMs, for which literature has identified seven types of latent interactions based on the specific components of the moderator and focal variable that constitute the latent interaction (Preacher et al., 2016). For example, a within-level interaction is formed using the within part of a moderator located at level 1 and the within part of a level 1 focal predictor (i.e., type A1 in Preacher et al., 2016). Both components that form the interaction are located at the within level, so a within-level latent interaction is formed, but between-level (i.e., type C in Preacher et al., 2016) and cross-level interactions (i.e., type A2 in Preacher et al., 2016) are also possible (Asparouhov & Muthén, 2020; Cox et al., 2023; Preacher et al., 2016).

### Measurement models

We utilize multilevel factor models to separate the within- and between-level components. For example, a latent focal predictor ${\eta }_{X}$ is decomposed into ${\eta}_{X}^{B}$ and ${\eta }_{X}^{W}$ such that

1 $${\text{X}}_{ij}={{\mu}_{{X}_{j}}+\Lambda}_{X}^{B}{\eta}_{{X}_{j}}^{B}+{\Lambda}_{X}^{W}{\eta }_{{X}_{ij}}^{W}+{\varepsilon }_{{X}_{j}}^{B}+{\varepsilon}_{{X}_{ij}}^{W}$$

In the multilevel factor model *i* and *j* index the individuals and clusters, respectively, ${X}_{ij}$ represents the indicators for the latent variable, the latent variable has a between-level component represented by ${\eta }_{{X}_{j}}^{B}$ and a within-level component ${\eta }_{{X}_{ij}}^{W}$, ${\Lambda }_{X}^{B}$ and ${\Lambda }_{X}^{W}$ are the corresponding factor loadings*,* ${\mu }_{{X}_{j}}$ denotes the intercepts for each cluster, and ${\varepsilon }_{{X}_{j}}^{B}$ and ${\varepsilon }_{{X}_{ij}}^{W}$ are the between- and within-level error terms, where ${\varepsilon }_{{X}_{j}}^{B}\sim N(0,{\tau }_{X}^{2})$ and ${\varepsilon }_{{X}_{ij}}^{W}\sim N(0,{\sigma }_{X}^{2})$. The scale of the latent variables can be set by setting the first factor loading of both ${\eta }_{{X}_{j}}^{B}$ and ${\eta }_{{X}_{ij}}^{W}$ or assigning unit variance to the between- and within-level factors. We utilize the latter method, assigning unit variance to the between- and within-level factors. Similar formulations are utilized for any latent moderator (e.g., ${Z}_{ij}$) or outcome (e.g., ${Y}_{ij}$). A standard factor model is utilized for the latent variables located solely at the between level. For example, the measurement model for a moderator ${Z}_{j}$ located at the between level is

2 $${Z}_{j}={\Lambda}_{Z}^{B}{\eta }_{{Z}_{j}}^{B}+{\varepsilon }_{{Z}_{j}}^{B}$$

with terms and notation similar to the multilevel factor model.

### Structural models

Let us begin with a 1×(1–1) design which reflects a level 1 moderator (${Z}_{ij}$), level 1 focal variable (${X}_{ij}$), and level 1 outcome (${Y}_{ij}$) such that ${Z}_{ij}$×(${X}_{ij}-{Y}_{ij}$). For the within-level latent interaction, ${\eta }_{{Z}_{ij}}^{W}$ moderates the effect of ${\eta}_{{X}_{ij}}^{W}$ on ${\eta}_{{Y}_{ij}}^{W}$, such that the model

for the outcome is

3 $$\begin{array}{lc}{\eta}_{{Y}_{ij}}^{W}={\beta}_{0}+{\beta}_{1}{\eta}_{{X}_{ij}}^{W}+{\beta}_{2}{\eta}_{{Z}_{ij}}^{W}+{\beta}_{3}{\eta}_{{X}_{ij}}^{W}{\eta}_{{Z}_{ij}}^{W}+{\varepsilon}_{ij}^{W}\\ {\eta}_{{Y}_{j}}^{B}={\gamma}_{0}+{\gamma}_{1}{\eta}_{{X}_{j}}^{B}+{\gamma}_{2}{\eta }_{{Z}_{j}}^{B}+{u}_{j}^{B}\end{array}$$

where

4 $$\begin{array}{lc}{\varepsilon}_{ij}^{W}\sim N(0,{\sigma}_{{Y}_{|}}^{2})\\ {u}_{j}^{B}\sim N(0,{\tau}_{{Y}_{|}}^{2})\end{array}$$

**Fig. 1:**
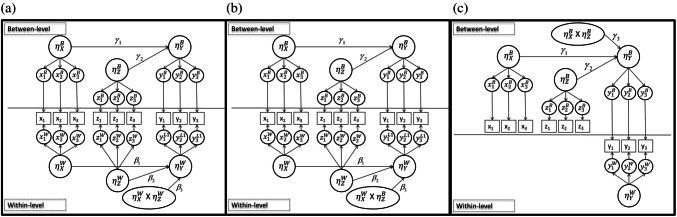
Conceptual representation of MLSEM with **a** within-level, **b** cross-level, and **c** between-level latent interactions

The ${\beta}_{0}$ term is the intercept with other $\beta$ coefficients capturing relationships between latent predictors and the outcome at the within level. At the between level, ${\gamma}_{0}$ is the intercept with other $\gamma$ coefficients capturing relationships between latent predictors and the outcome. For each coefficient associated with a latent predictor, we assume a nonrandom slope where ${\varepsilon}_{ij}^{W}$ is the within-level residual and ${u}_{j}^{B}$ is the between-level residual. The residuals follow a normal distribution with a mean of 0 and conditional variances of ${\sigma}_{{Y}_{|}}^{2}$ and ${\tau}_{{Y}_{|}}^{2}$ at the respective within and between levels. The ${\beta}_{3}$ coefficient captures the within-level interaction effect, as both predictors of the interaction (i.e., ${\eta}_{{X}_{ij}}^{W}$ and ${\eta }_{{Z}_{ij}}^{W}$) are located at the within level (see Fig. 1a).

For the cross-level interaction, ${\eta}_{{Z}_{j}}^{B}$ moderates the effect of ${\eta}_{{X}_{ij}}^{W}$ on ${\eta}_{{Y}_{ij}}^{W}$, such that the model is

5 $$\begin{array}{lc}{\eta}_{{Y}_{ij}}^{W}={\beta}_{0}+{\beta}_{1}{\eta}_{{X}_{ij}}^{W}+{\beta}_{2}{\eta}_{{Z}_{ij}}^{W}+{\beta}_{3}{\eta}_{{X}_{ij}}^{W}{\eta}_{{Z}_{j}}^{B}+{\varepsilon}_{ij}^{W}\\ {\eta}_{{Y}_{j}}^{B}={\gamma}_{0}+{\gamma}_{1}{\eta}_{{X}_{j}}^{B}+{\gamma}_{2}{\eta}_{{Z}_{j}}^{B}+{u}_{j}^{B}\end{array}$$

Distributional assumptions for the residual terms remain the same (see Eq. 4), as do interpretations of the other terms and notation (see Eq. 3). The ${\beta}_{3}$ coefficient again captures the interaction effect. However, the product term now includes the between-level component of the moderator (${\eta}_{{Z}_{j}}^{B}$) and the within-level component of the focal variable (${\eta}_{{X}_{ij}}^{W}$). Conceptually, the components of the interaction are located at different levels, so we have a cross-level interaction (see Fig. 1b).

Switching to a 2 × (2–1) design, the moderator and focal predictor are located entirely at level 2, allowing us to drop the *i* from the notation. For this type of latent interaction, ${\eta }_{{Z}_{j}}^{B}$ influences the relationship between ${\eta}_{{X}_{j}}^{B}$ and ${\eta }_{{Y}_{j}}^{B}$, such that the model is

6 $$\begin{array}{lc}{\eta}_{{Y}_{ij}}^{W}={\beta }_{0}+{\varepsilon}_{ij}^{W}\\ {\eta}_{{Y}_{j}}^{B}={\gamma}_{0}+{\gamma}_{1}{\eta}_{{X}_{j}}^{B}+{\gamma}_{2}{\eta}_{{Z}_{j}}^{B}+{\gamma}_{3}{\eta}_{{X}_{j}}^{B}{{\eta}_{{Z}_{j}}^{B}+u}_{j}^{B}\end{array}$$

Again, the distributional assumptions for the residual terms remain the same (see Eq. 4), and the interpretation of other terms and notation parallels previous expressions (see Eq. 3). The coefficient associated with the interaction is now ${\gamma}_{3}$ and includes the between-level moderator component (${\eta}_{{Z}_{j}}^{B}$) and the between-level focal variable component (${\eta}_{{X}_{j}}^{B}$). These components are both located at the between level, so we have a between-level interaction (see Fig. 1c). In summary, we have a set of latent interaction models in which the location (i.e., level) of the interaction changes. In each model the predictors are multivariate normal and the outcome is model-implied non-normal.

## Structural-after-measurement approaches

Broadly, SAM approaches separate estimation of SEMs into measurement and structural components. In contrast, full-information estimators (e.g., Bayes) simultaneously estimate the measurement and structural model parameters that form a complete SEM (Rosseel & Loh, 2024). Prior literature has suggested at least four advantages to the SAM approach:

1. Estimates that are more robust to local model misspecifications,
2. Improved model convergence in small samples,
3. Reduced (finite) bias under correctly specified models, and
4. Reduced computational burden for latent interaction (e.g., Cox & Kelcey, 2021; Rosseel & Loh, 2024; Rosseel et al., 2025).

In our context involving SEMs with latent interactions, complex data structure, and limited sample sizes, advantages (2) through (4) are particularly pertinent.

A key advantage of SAM approaches is their adaptability and scalability. Separation of the measurement and structural models accommodates model and data complexity while maintaining simplicity in estimation. For example, the computational demands of SAM estimators grow linearly with complexity (e.g., when adding several latent interactions). By contrast, the computational demands of full-information estimators (e.g., Bayesian and ML estimators) tend to increase exponentially with model complexity. For example, conventional ML estimation with latent interactions quickly becomes infeasible in multilevel settings with two or more latent interactions, because it requires integration that scales exponentially with the addition of latent interactions. Bayesian estimation is more feasible with complex models, but the computational demands, particularly with small samples, frequently introduce estimation problems that result in poor mixing, non-convergence, or severely biased estimates.

Prior literature has developed several variations for SAM estimation (Devlieger et al., 2016; Rosseel & Loh, 2024). Wall and Amemiya (2000) developed a two-stage method of moments approach that is applicable to linear and nonlinear SEMs. There are also several factor score regression approaches that introduce some type of correction or method to avoid biased parameter estimates in the structural model (Devlieger et al., 2016; Hoshino & Bentler, 2013; Skrondal & Laake, 2001). For example, Skrondal and Laake (2001) utilized Bartlett factor scores for endogenous latent variables to avoid bias. Ng and Chan (2020) simplified and extended a similar approach to accommodate latent higher-order terms, using Bartlett factor scores for latent response variables but regression factor scores for latent predictors. More recently, Rosseel and Loh (2024) established a highly flexible framework for SAM estimation that funnels into two different types of methods: local SAM and global SAM. Global SAM estimates the measurement model parameters for all latent variables and then fixes them for estimation of the structural model, while local SAM approaches estimate individual measurement models for latent variables and then use those results and various corrections to estimate structural parameters (see Rosseel & Loh, 2024, for a more comprehensive description). The local SAM approach of Rosseel and Loh (2024) has been extended using the Kronecker (or tensor) product to accommodate latent interactions and quadratic terms (Rosseel et al., 2025).

Our analysis focuses on a type of local SAM estimator—SAM-Croon. In our application of SAM-Croon, we use factor score regression with a correction for bias stemming from ignoring the uncertainty in the measurement models (e.g., Croon, 2002; Cox & Kelcey, 2021; Cox et al., 2023), although other iterations of SAM-Croon and local SAM estimation avoid prediction of factor scores altogether and focus on covariance of the indicators (e.g., Devlieger & Rosseel, 2020; Rosseel et al., 2025). Our approach can be conceptually described in a four-step process. First, factor models (single or multilevel) are estimated for each latent variable using ML and are used to predict factor scores. In the second step, a variance–covariance matrix of factor scores (and any observed variables) is estimated. Third, the variance–covariance matrix produced in the second step is corrected using results from the measurement models estimated in the first step to account for the uncertainty in the step 1 measurement models. Conceptually, when regression-based factor scores are predicted, the measurement models provide an estimate of latent variable unreliability that is used to adjust the covariance matrix in proportion to this unreliability. In the fourth and final step, the corrected variance–covariance matrix is used to estimate the structural or path model.

The nature of the correction in the third step is often what distinguishes different SAM approaches. SAM-Croon uses a method of moments type correction to adjust the predicted variance–covariance matrix to account for the attenuation stemming from unreliability in the latent variable factor scores (Croon, 2002). The process conceptually parallels disattenuation formulas from classical test theory for correlations between two variables measured with error. The factor models capture the unreliability of the latent variable measures, and the correction adjusts the covariance of the factor scores as a function of this unreliability. The corrections take on slight variations based on the variables and level involved in the correlation. For example, in MLSEMs, different corrections are applied at the between and within levels, and corrections also differ according to whether the correlation involves an observed or latent variable or both.

Our SAM approaches rely on the covariance of factor scores at the between and within levels (see Eq. 1). The clustering of observed indicators must be accounted for in the SAM-Croon corrections that involve a within-level latent variable that varies within and between levels (i.e., within- and cross-level interactions). Let us again focus on a focal predictor ${\eta}_{X}$ and its between-level factor scores. With three indicators, the between-level factors scores are found using

7 $${\widetilde{X}}_{j}^{B}={A}_{{x}_{1}}^{B}{x}_{1}^{B}+{A}_{{x}_{2}}^{B}{x}_{2}^{B}+{A}_{{x}_{3}}^{B}{x}_{3}^{B}$$

The predicted factor scores for each cluster are represented with ${\widetilde{X}}_{j}^{B}$, between-level components of the focal predictor indicators are ${x}^{B}$, and the between-level factor score matrices are ${A}_{x}^{B}$. There are several alternative approaches for predicting the between- or cluster-level values of indicators. We use a univariate cluster means approach to estimate the between-level indicator components. Cluster means is a simple estimate of the cluster-level components of the indicators using the observed cluster-specific means. However, alternative approaches include, for example, the use of empirical Bayes predictions of indicators or level-specific covariances that avoid estimating the between-level component of the indicator altogether.

Methods for tracking standard errors or confidence intervals for path coefficients, interactions, or more complex effects (e.g., mediation or moderated mediation) from SAM approaches is an area of active research. Several recent and forthcoming papers have developed and investigated analytic and bootstrap-based methods (e.g., Cox & Kelcey, 2021; Dhaene, & Rosseel, 2023; Kelcey, 2019; Kelcey et al., 2021; Rosseel & Loh, 2024; Savalei & Rosseel, 2021; Rosseel et al., 2025). For example, prior research has demonstrated that bootstrap methods can generate asymmetric confidence intervals and inferences for SAM estimates using a sample-with-replacement approach that repeats the SAM estimation process. Using, for example, 1,000 of these bootstrap replications, confidence intervals can be developed at 2.5% and 97.5% of the distribution of the estimate or effect. With asymmetric confidence intervals, inferences can be drawn about the significance of each estimate or effect (see Cox & Kelcey, 2021, for example and R code).

### Croon corrections for multilevel latent interactions

For SAM approaches with SEMs that include latent interactions, we utilized the product of the factor scores for each latent variable that comprised the interaction term to estimate the latent interaction. The covariance between this interaction term (product of factor scores) and the factor scores of the latent outcome is corrected in the SAM-Croon approach using implied unreliability from the measurement models of all latent variables involved in the covariance term (i.e., both variables that form the interaction and those that form the outcome). For example, the correction for covariance of the within-level interaction and outcome presented in Fig. 1a would be (Cox et al., 2023)

8 $$\text{cov}({\eta}_{Z}^{W}{\eta}_{X}^{W},{\eta}_{Y}^{W})=\frac{\text{cov}({\widehat{\eta}}_{Z}^{W}{\widehat{\eta}}_{X}^{W},{\widehat{\eta}}_{Y}^{W})}{{A}_{Z}^{W}{A}_{X}^{W}{\Lambda}_{Z}^{W}{\Lambda}_{X}^{W}{\Lambda}_{Y}^{W}{A}_{Y}^{W}}$$

Recall that $$\Lambda$$ represents the corresponding factor loading matrices for the latent variables. Using superscript, we denote the factor loading matrices at the within ($${\Lambda}_\cdot^{W}$$) and between levels ($${\Lambda}_\cdot^{B}$$). Similarly, $$A$$ represents the corresponding factor score matrices for the latent variables at the within ($${A}_\cdot^{W}$$) and between levels ($${A}_\cdot^{B}$$). Covariance terms that include components from the between level require an additional term in the denominator ($${R}^{B})$$ to adjust for the unreliability of indicator cluster means. The $${R}^{B}$$ term captures the vector of mean indicator reliabilities (see Cox et al., 2023, for details on all corrections).

In addition, the variance of the factor score interaction term was estimated using the formula for variance of the product of two normal variables (Bohrnstedt & Goldberger, 1969). The estimated variance of the factor score interaction term from the within-level interaction example would be found using

9 $$\text{var}({\eta}_{Z}^{W}{\eta}_{X}^{W})=\text{var}({\eta}_{Z}^{W})\text{var}({\eta}_{X}^{W})+\text{cov}{({\eta}_{Z}^{W},{\eta}_{X}^{W})}^{2}$$

This expression relies on the multivariate normality of the latent variables. Recent literature has demonstrated that some SAM approaches are more robust to certain violations of this multivariate normality assumption (Rosseel et al., 2025).

## Multilevel SEM simulation study methods

We conducted a series of three Monte Carlo simulation studies to compare SAM and Bayesian estimation for MLSEMs with three different latent interactions (i.e., within-, between-, and cross-level latent interactions) across limited sample size conditions. To consider the relative performance of estimators and further explicate their performance, we included a SAM approach with uncorrected factor scores (i.e., SAM-FS), the SAM approach with the aforementioned Croon correction (SAM-Croon), Bayesian estimation with naïve (i.e., noninformative) priors (BAYES), and Bayesian estimation with informative priors (BAYES-IN). Estimator performance was evaluated using measures of accuracy, efficiency, and feasibility.

### Data generation

Data were generated based on the structural and measurement models detailed above and illustrated in Fig. 1 (see Table 1 for specific values). This includes a covariance between *X* and *Z* at the within and between levels of 0.25. We generated 500 datasets and varied sample size in a fully crossed design with ${n}_{2}=\text{30, 60},\ \text{and}\ 90$ clusters and ${n}_{1}=20\text{and}50$ individuals per cluster. These sample size conditions reflect previous literature examining estimation of MLSEMs with limited sample size (e.g., Cox et al., 2023; Smid & Rosseel, 2020) and many practical limitations in substantive research (e.g., Schochet, 2011). Measurement models used three indicators per latent variable, with factor loadings set at 1.0. All coefficients associated with individual variables were set to 0.4 (e.g., ${\beta}_{1}=0.4$ and ${\gamma}_{1}=0.4$), with the coefficient representing the interaction set to 0.2 (e.g., ${\beta}_{3}=0.2$ and ${\gamma}_{3}=0.2$) and intercepts set to 0 (e.g., ${\beta}_{0}=0.0$ and ${\gamma}_{1}=0.0$). These coefficients are all scaled as standardized regression coefficients.

**Table 1:** Simulation study fixed population model parameters

Model	Measurement			Structural				
	Factor loadings	Intercepts	Variance	Covariance	Intercepts	Path coefficients	Interaction	Variance
Full	$\Lambda=1$	$\mu=0$	${\sigma}^{2}=0.8$ ${\tau}^{2}=0.2$	${Cov}^{W}(X,Z)=0.25$ ${Cov}^{B}(X,Z)=0.25$	${\beta}_{0}=0$ ${\gamma }_{0}=0$	${\beta}_{\text{1,2}}=0.4$ ${\gamma}_{\text{1,2}}=0.4$	${\beta}_{3}=0.2$ OR ${\gamma }_{3}=0.2$	${\sigma}^{2}=0.8$ ${\tau}^{2}=0.2$
Partial								
treat	$\Lambda=1$	$\mu=0$	${\sigma }^{2(t)}=0.8$ ${\tau }^{2(t)}=0.2$	${Cov}^{W}\left(M,Z\right)=0.0$ ${Cov}^{B}(M,Z)=0.0$	${a}^{(t)}=0.5$ ${\mu}_{0}^{(t)}=0.7$	${B}^{\left(t\right)}=0.4$ ${b}_{1}^{\left(t\right)}=0$ ${\xi}_{1}^{(t)}=0.2$	${\xi}_{2}^{(t)}=0.15$	${\sigma}^{2(t)}=0.8$ ${\tau }^{2(t)}=0.2$
Control	$\Lambda=1$	$\mu=0$	${\sigma}^{2(c)}=1$		${a}^{(c)}=0$ ${\mu}_{0}^{(c)}=0$	${b}_{1}^{(c)}=0.4$		${\sigma}^{2(c)}=1$

The residual variance decomposition for latent variables was set using ${\sigma }^{2}=0.8$ and ${\tau }^{2}=0.2$. A matching structure was used to set the variance decomposition of the three observed indicators associated with each latent variable. The expected composite reliability (Omega) of the between-level components of the latent variables is 0.94 and 0.79 for the within-level components based on the error variance, number of indicators, and factor loadings. To ensure that these conditions were adequate for an initial investigation of estimator performance, we conducted a supplemental simulation that varied factor loadings and residual variance decomposition in MLSEMs that included a within-level and a cross-level latent interaction (see Supplemental Materials on study OSF storage site).

Based on the true population values, the overall variance explained in the outcome on the within level for the MLSEM with a within-level interaction is approximately 33% and 66% at the between level. Approximately 3% of the explained variance in the outcome at the within level is owed to the within-level interaction. The MLSEM with a cross-level interaction explained the same amounts of variance in the outcome, with the cross-level interaction again explaining around 3% of the outcome variance at the within level. The MLSEM with a between-level interaction explained 72% of the variance in the outcome at the between level, with 8% of the explanation owing to the between-level interaction. The amount of variance explained in the outcome is going to vary across substantive contexts, with the amounts in our simulation study considered high in some contexts but typical in others. For example, in education, when standardized achievement assessments are used, prior literature has suggested that models including pretest assessments of achievement typically explain large proportions of outcome variance (e.g., Hedges and Hedberg, 2007, 2014).

We have limited this comparison study to continuous data, although SAM approaches that accommodate other variable types are in development (Kuha & Bakk, 2024). Second, we assume the latent variables in the model including those in the interaction term follow a normal distribution. Adapting SAM methods as well as other estimation approaches for SEMs to non-normal latent variables is also an active area of research (e.g., Rosseel et al., 2025). While recent literature has suggested that parameter estimates and model fit are often robust to non-normality in latent variables for linear SEM and other linear mixed-effects models (e.g., Jobst et al., 2021; Schielzeth et al., 2020; Shi & Maydeu-Olivares, 2020; Xia & Yang, 2019), these results should not be blindly applied to nonlinear SEMs that include multiple indicator measurement models. Proper investigation of the robustness of the SAM methods with nonlinear SEMs to violations of this normality assumption are an important consideration. Recent work has demonstrated that the SAM-Croon approach can be susceptible to convergence and estimation issues when latent variables follow a non-normal distribution (Rosseel et al., 2025). Our analyses specifically examine the comparative performance of SAM-Croon and Bayesian approaches when the normality assumption is met. It is also important to note that SAM methods are flexible and can be readily tailored to a variety of settings. Given the diversity in SAM methods, issues identified with SAM-Croon do not broadly apply to all SAM approaches. For example, although SAM-Croon can underperform in non-normal settings, adaptations of the local SAM approach developed in Rosseel et al. (2025) demonstrated strong performance even with latent variables that follow normal and non-normal distributions.

### Analysis and estimators

Data generation and analyses were completed using a combination of Mplus 8.11 (Muthén & Muthén, 1998-2021) and R (R Development Core Team, 2019). Data generation and the aggregation of results was conducted in R, while model estimation was performed in Mplus. The *MplusAutomation* package in *R* was used to bridge software packages (Hallquist & Wiley; 2018).

We excluded conventional full-information ML estimation in our comparison study due to feasibility issues. ML estimation of latent interactions requires (potentially high-dimensional) numerical integration. Within the context of multilevel settings, the inclusion of several latent variables and interactions typically renders numerical integration computationally challenging and sometimes practically infeasible (Klein & Moosbrugger, 2000). For example, Asparouhov & Muthén, (2020) found that ML estimation of simple MLSEMs with a single latent interaction often produced non-convergence rates of 100%, while Cox et al. (2023) found high convergence failure rates and many implausible values when estimates were produced. However, typical ML estimation is still suitable and utilized for the separate measurement and structural models in both SAM approaches (i.e., uncorrected factor scores and with Croon’s correction).

For both Bayesian estimators we used the Markov chain Monte Carlo (MCMC) algorithm with a maximum of 20,000 iterations to reach a potential scale reduction (PSR) convergence criterion of < 1.1 (see Gelman & Rubin, 1992, and Asparouhov & Muthén, 2010). The first Bayesian approach (BAYES) employed naïve priors under Mplus default settings (see Muthén & Muthén, 1998-2021) for all measurement and structural parameters (e.g., path coefficients, factor loadings, and variance parameters). The second Bayesian approach (BAYES-IN) employed informative priors for all estimated factor loadings and path coefficients such that $N(pop,1)$, where *pop* is set using the true value used in data generation. Priors for variance of the latent outcome variable were also set for BAYES-IN using the inverse-gamma distribution (*IG*) with the parameter values set to reflect population true variance values. Recall that the scale of the latent moderator and focal predictor were set to 1, so setting values for these priors was not applicable. Again, to ensure these conditions were adequate, we examined the performance of BAYES-IN with misspecified priors as part of our supplemental simulation (see Supplemental Materials).

### Estimator comparison outcomes

We assessed estimator performance using three general criteria: accuracy, efficiency, and feasibility. For accuracy, we tracked bias in specific coefficient estimates including the coefficient associated with the interaction term. We used the standard deviation of these estimates across simulation runs to capture sampling variability and assess estimator efficiency. In addition, we report root mean square error to consider the combination of accuracy and sampling variability indicated separately by calculations of bias and the standard deviation of the parameter estimates. To assess the feasibility of each estimator, we captured model convergence rate and time to convergence.

#### Convergence

Non-convergence can often be viewed as a nuisance in simulations studies, and results are typically tabulated on those samples that have converged. Non-convergence, however, represents a pressing issue in applied research, because with only a single dataset available it can fundamentally undermine or substantially limit the empirical evidence produced. This issue is particularly relevant for studies examining interactions among latent variables, because they are notoriously difficult to estimate.

We defined model convergence as an estimator’s ability to produce plausible results given convergence criteria. For SAM approaches, model non-convergence often indicated that the estimator failed to produce results (i.e., coefficient estimates) due to non-positive definite covariance matrices or variance estimates on the boundary (zero). These issues have been investigated, with appropriate solutions now available (e.g., the lambda correction noted in Bogaert et al., 2022). For the Bayesian approaches, model non-convergence was defined as failure to meet potential scale reduction values less than 1.1 (PSR < 1.1) by 20,000 iterations.

#### Bias

We began with a set of population parameters (see Table 1) used to generate data. For example, ${\Theta}_{xz}^{Pop}$ represents the true population parameter for the coefficient associated with the interaction term. For bias in specific interaction coefficients and effects, we found the difference between the true population parameter and the mean of estimates from each estimator across replications, which we represent with ${\overline{\widehat{\Theta}} }_{.}$. For example, the means of estimates for the path coefficient associated with the interaction term (*xz*) were found using

10 $${\overline{\widehat{\Theta}}}_{xz}=\sum\nolimits_{i=1}^{R}\frac{{\widehat{\Theta}}_{xz,i}}{R}$$

Here, ${\widehat{\Theta }}_{xz,i}$ are the estimates for the coefficient associated with the interaction term, which are then averaged across replications $(i=\text{1,2},...,R)$. Bias for estimates of this interaction term would then be calculated using

11 $${Interaction\ coefficient\ bias ={\overline{\widehat{\Theta}} }_{xz}-\Theta}_{xz}^{Pop}$$

For a broader, more summative measure of bias, we found the average absolute bias across all coefficient estimates at a given level (i.e., within level or between level). We began with absolute bias from each coefficient estimate found by contrasting the estimates produced by each approach and empirical estimates from the true model (${\overline{\widehat{\Theta}} }_{x}-{\overline{\Theta} }_{x}^{True}$). The true empirical estimates use the exact latent variable values (i.e., prior to indicator variable generation). Specifically, we found the average coefficient estimate for the different coefficients by estimator approach and then subtracted the average true empirical estimate. To find the average coefficient estimate for each coefficient by estimator we used

12 $${\overline{\widehat{\Theta}} }_{x}=\sum\limits_{i=1}^{R}\frac{{\widehat{\Theta}}_{xi}}{R}$$

where *x* represents a coefficient from the model and *R* denotes the simulation replications $(i=\text{1,2},...,R)$. The true model average coefficients were found using a similar approach,

13 $${\overline{\Theta } }_{x}^{True}=\sum\limits_{i=1}^{R}\frac{{\Theta }_{xi}^{True}}{R}$$

The average absolute bias is then the average of the differences between these terms such that

14 $$\text{Average absolute bias}=\sum\limits_{i=1}^{n}\frac{\left\vert{\overline{\widehat{\Theta}} }_{{x}_{i}}-{\overline{\Theta}}_{{x}_{i}}^{True}\right\vert}{n},$$

where *n* is the number of applicable coefficients estimated in the model.

#### Efficiency

Along with bias measures, we report the standard deviation of the interaction coefficient estimates and the average standard deviation of other parameter estimates to indicate estimator sampling variability. These descriptions of estimator sampling variability indicate the precision of estimates and therefore the efficiency of each estimator. To combine considerations of bias and efficiency we report the average root mean square error (RMSE). We squared bias in the coefficient estimates, added the variance of the estimate across replications, and found the square root of this sum. We then averaged these RMSE values and report an average RMSE across all between- and within-level estimates (when necessary) for each estimator.

## Results

We now share results from the series of three Monte Carlo simulation studies comparing estimator performance with MLSEMs that included latent interactions. Results are organized by comparison criteria (i.e., model convergence rate, bias, and efficiency). We limit this discussion to results across latent interaction type and sample size condition as results for conditions that included reduced factor loadings, increased residual variance at the between level, and misspecified priors condition for BAYES-IN were similar (see Supplemental Materials).

Before presenting results, it is important to note that our initial results involving Bayesian estimation of an MLSEM with a cross-level interaction included severely biased estimates of between-level factor loadings, path coefficients, variance, and residual variance involving the focal predictor. After investigating, and with the help of Mplus support, a bug was identified in Mplus version 8.11. This will be corrected in the next version of Mplus, but support was able to provide an immediate temporary correction that involved adding artificial direct effects for the indicators of the between-level factor involved in the cross-level interaction (see x21 x22 x23 on x21@0 in R code). We tested this correction with a variety of models and with a variety of sample sizes and found that it remedied the issue.

### Convergence

Neither SAM nor Bayesian estimators experienced convergence issues in the MLSEM with a within-level latent interaction (see Table 2). Acceptable performance was also found for estimation of the SEM with a between-level latent interaction. SAM-Croon did produce some cases of non-convergence with this model at the smallest sample size condition, but the failure rate never exceed 8%. Bayesian approaches for the SEM with a cross-level interaction produced the most notably poor convergence results, particularly Bayesian estimation with naïve priors. For BAYES, convergence criteria were not met in more than 50% of the simulation runs in most sample size conditions. Inclusion of informative priors (BAYES-IN) substantially improved convergence, but failure rates still exceeded 25% in four of the six conditions. The computational issues for Bayesian approaches were also reflected in longer time to convergence for estimates of the model with a cross-level interaction. We did assess the effect of increasing the maximum number of iterations (e.g., from 20,000 to 100,000) for the Bayesian approaches and found it improved convergence rates. However, this led to considerable increases in time to model convergence. To summarize, both SAM and Bayesian approaches performed well in terms of convergence rates for SEMs with within- and between-level interactions, but Bayesian approaches struggled to converge under typical criteria when estimating SEMs with cross-level interactions.

**Table 2:** Convergence failure rate for various estimators of a multilevel SEM with various latent interactions

		Latent interaction											
Sample size		Within-level				Cross-level				Between-level			
${n}_{2}$	${n}_{1}$	Bayes	BayesIN	FS	Croon	Bayes	BayesIN	FS	Croon	Bayes	BayesIN	FS	Croon
30	50	0%	0%	0%	<1%	63%	27%	0%	<1%	<1%	0%	0%	4%
60		0%	0%	0%	0%	74%	35%	0%	0%	0%	0%	0%	3%
90		0%	0%	0%	0%	67%	30%	0%	0%	0%	0%	0%	1%
30	20	0%	0%	0%	2%	44%	5%	0%	2%	1%	0%	0%	8%
60		0%	0%	0%	0%	53%	7%	0%	<1%	0%	0%	0%	3%
90		0%	0%	0%	0%	58%	27%	0%	<1%	0%	0%	0%	1%

*Note:* Bayesian estimators included naïve priors (Bayes) and informative priors (Bayes-IN), FS is the SAM uncorrected factor score regression approach, and Croon is the SAM approach with Croon-based correction of factor score regression

### Bias

We begin by focusing on coefficient estimates for the latent interactions (see Table 3), then consider the average bias for the estimation approaches at each level. Overall, the estimators performed well in terms of bias for estimates of the coefficient associated with the different latent interactions, with two notable exceptions. First, the naïve Bayesian estimation approach for the SEM with a cross-level interaction consistently produced substantially inaccurate estimates and had the greatest variability in these estimates based on the *SD* across simulation runs. These results included all estimates that the naïve Bayesian approach was able to produce including those when the PSR still exceeded 1.1. Second, SAM-FS consistently underestimated the interaction coefficient. This was expected, as SAM-FS disregards the measurement error associated with the latent variables, allowing it to function with limited sample sizes but also producing biased estimates.

**Table 3:** Bias in interaction coefficient effect estimates with standard deviation of estimates for multilevel structural equation models with within-level, cross-level, and between-level type latent interactions

*Est.*	*Sample size*		Estimator							
	${n}_{2}$	${n}_{1}$	Bayes		BayesIN		FS		Croon	
Within	30	50	0.00	(0.04)	0.00	(0.04)	−0.03	(0.03)	−0.01	(0.04)
	60		0.00	(0.02)	0.00	(0.02)	−0.03	(0.02)	−0.01	(0.03)
	90		0.00	(0.02)	0.00	(0.02)	−0.04	(0.02)	−0.01	(0.02)
	30	20	0.00	(0.06)	0.00	(0.06)	−0.04	(0.05)	−0.03	(0.06)
	60		0.00	(0.04)	0.00	(0.04)	−0.04	(0.03)	−0.03	(0.04)
	90		0.00	(0.03)	0.00	(0.03)	−0.04	(0.03)	−0.03	(0.03)
Cross	30	50	−0.26	(0.26)	0.03	(0.11)	−0.04	(0.04)	−0.01	(0.04)
	60		−0.05	(0.21)	0.02	(0.07)	−0.04	(0.03)	−0.01	(0.03)
	90		−0.12	(0.24)	0.04	(0.08)	−0.04	(0.02)	−0.01	(0.02)
	30	20	−0.16	(0.25)	0.02	(0.08)	−0.05	(0.05)	−0.02	(0.06)
	60		−0.03	(0.16)	0.02	(0.06)	−0.04	(0.03)	−0.01	(0.04)
	90		−0.13	(0.20)	0.02	(0.05)	−0.04	(0.03)	−0.01	(0.04)
Between	30	50	0.04	(0.23)	0.04	(0.21)	−0.05	(0.16)	−0.04	(0.21)
	60		0.01	(0.12)	0.01	(0.12)	−0.04	(0.11)	−0.02	(0.14)
	90		0.01	(0.09)	0.01	(0.09)	−0.03	(0.08)	−0.01	(0.11)
	30	20	0.03	(0.26)	0.03	(0.21)	−0.06	(0.16)	−0.05	(0.21)
	60		0.01	(0.13)	0.00	(0.13)	−0.05	(0.10)	−0.03	(0.14)
	90		0.01	(0.10)	0.01	(0.10)	−0.04	(0.09)	−0.01	(0.11)

An interesting pattern in bias emerged from BAYES-IN estimates of the coefficient associated with the latent interaction. Even with correctly specified priors centered on the true population coefficient, BAYES-IN still produced an inflated estimate (around 10–20% relative bias) for cross-level interactions and likewise for the between-level interactions. Conversely, SAM-Croon estimates of the coefficients associated with the latent interaction tended to be underestimated as sample size was reduced. When directly comparing SAM-Croon and Bayesian approaches, we found minor differences in bias results across the different types of latent interactions. Bayesian estimates of the within-level interaction were very accurate even at the smallest sample size condition. Estimates for the within-level interaction from SAM-Croon were acceptable but were downwardly biased as the ${n}_{1}$ sample size decreased. SAM-Croon did perform well with the cross-level interaction. Naïve Bayes severely underestimated this type of latent interaction, and BAYES-IN produced its most inflated estimates. Lastly, the latent interaction at the between level, C, was the most difficult to estimate across approaches, but BAYES, BAYES-IN, and SAM-Croon still produced acceptable results in most conditions. This result aligns with expectations, as the between-level interaction is most reliant on ${n}_{2}$ sample sizes that were restricted to values of only 30, 60, and 90.

Beyond the specific coefficient estimate for the interaction term, we also compared bias in estimates from the SAM and Bayesian approaches across all coefficients in the model (see Table 4). We calculated the average bias for each estimator for coefficients at the between level and, when necessary, also at the within level. When considering all estimates, SAM-Croon and BAYES-IN performed well and very similarly. As noted, the only estimator with a persistent bias issue was naïve Bayes with the SEM that included a cross-level latent interaction.

**Table 4:** Average bias and average standard deviation of estimates across coefficients at the within and between levels for multilevel structural equation models with within-level, cross-level, and between-level type latent interactions

*Interaction (level)*	*Sample size*		Average absolute bias across all coefficients							
	${n}_{2}^{(t)}$	${n}_{1}^{(t)}$	Bayes		BayesIN		FS		Croon	
Within (B)	30	50	0.05	(0.15)	0.03	(0.14)	0.06	(0.12)	0.02	(0.14)
	60		0.01	(0.10)	0.00	(0.09)	0.05	(0.09)	0.01	(0.09)
	90		0.00	(0.08)	0.00	(0.08)	0.05	(0.07)	0.00	(0.08)
	30	20	0.03	(0.16)	0.02	(0.14)	0.07	(0.12)	0.01	(0.14)
	60		0.01	(0.11)	0.01	(0.10)	0.06	(0.09)	0.01	(0.10)
	90		0.00	(0.08)	0.00	(0.08)	0.06	(0.07)	0.01	(0.08)
Within (W)	30	50	0.00	(0.04)	0.00	(0.04)	0.05	(0.03)	0.01	(0.04)
	60		0.00	(0.03)	0.00	(0.03)	0.05	(0.02)	0.01	(0.02)
	90		0.00	(0.02)	0.00	(0.02)	0.05	(0.02)	0.01	(0.02)
	30	20	0.01	(0.06)	0.01	(0.06)	0.05	(0.05)	0.02	(0.06)
	60		0.00	(0.04)	0.00	(0.04)	0.05	(0.03)	0.02	(0.04)
	90		0.00	(0.03)	0.00	(0.03)	0.05	(0.03)	0.02	(0.03)
Cross (B)	30	50	*	(*)	0.05	(0.18)	0.06	(0.12)	0.02	(0.13)
	60		0.11	(0.26)	0.02	(0.14)	0.05	(0.09)	0.00	(0.10)
	90		0.13	(0.30)	0.01	(0.14)	0.05	(0.07)	0.00	(0.08)
	30	20	0.11	(0.33)	0.01	(0.18)	0.08	(0.12)	0.02	(0.14)
	60		0.05	(0.23)	0.02	(0.12)	0.06	(0.09)	0.00	(0.10)
	90		0.21	(0.29)	0.02	(0.11)	0.06	(0.07)	0.01	(0.08)
Cross (W)	30	50	0.09	(0.12)	0.01	(0.07)	0.05	(0.04)	0.01	(0.04)
	60		0.02	(0.09)	0.01	(0.05)	0.05	(0.03)	0.01	(0.03)
	90		0.04	(0.10)	0.02	(0.05)	0.05	(0.02)	0.01	(0.02)
	30	20	0.06	(0.13)	0.01	(0.07)	0.05	(0.05)	0.02	(0.06)
	60		0.02	(0.09)	0.01	(0.05)	0.05	(0.04)	0.02	(0.04)
	90		0.05	(0.10)	0.01	(0.04)	0.05	(0.03)	0.01	(0.03)
Between	30	50	0.04	(0.22)	0.04	(0.18)	0.06	(0.15)	0.03	(0.20)
	60		0.01	(0.12)	0.01	(0.12)	0.05	(0.10)	0.02	(0.13)
	90		0.01	(0.09)	0.01	(0.09)	0.04	(0.08)	0.01	(0.10)
	30	20	0.05	(0.25)	0.05	(0.19)	0.08	(0.15)	0.04	(0.20)
	60		0.02	(0.13)	0.02	(0.13)	0.06	(0.10)	0.02	(0.13)
	90		0.01	(0.10)	0.01	(0.10)	0.05	(0.09)	0.01	(0.11)

*Note:* Average bias and *SD* of estimates are separated by between level (B) and within level (W), with an asterisk (*) indicating that the estimator produced excessive average absolute bias (e.g., average absolute bias >1.00)

### Efficiency

First, consider the standard deviation of coefficient estimates for the interaction (see Table 3). Overall, the four estimators had similar performance, with issues paralleling those involving bias:

1. Naïve Bayes had large *SD* values with the SEM including the cross-level interaction;
2. By disregarding measurement error associated with the latent variables, SAM-FS often produced the most efficient estimates;
3. Sampling variability performance for all estimators generally converged as sample sizes increased (i.e., ${n}_{2}\ge 90$).

The results for average *SD* for all of the coefficient estimates in Table 4 follow very similar patterns to the interaction coefficient-specific *SD*s.

**Fig. 2:**
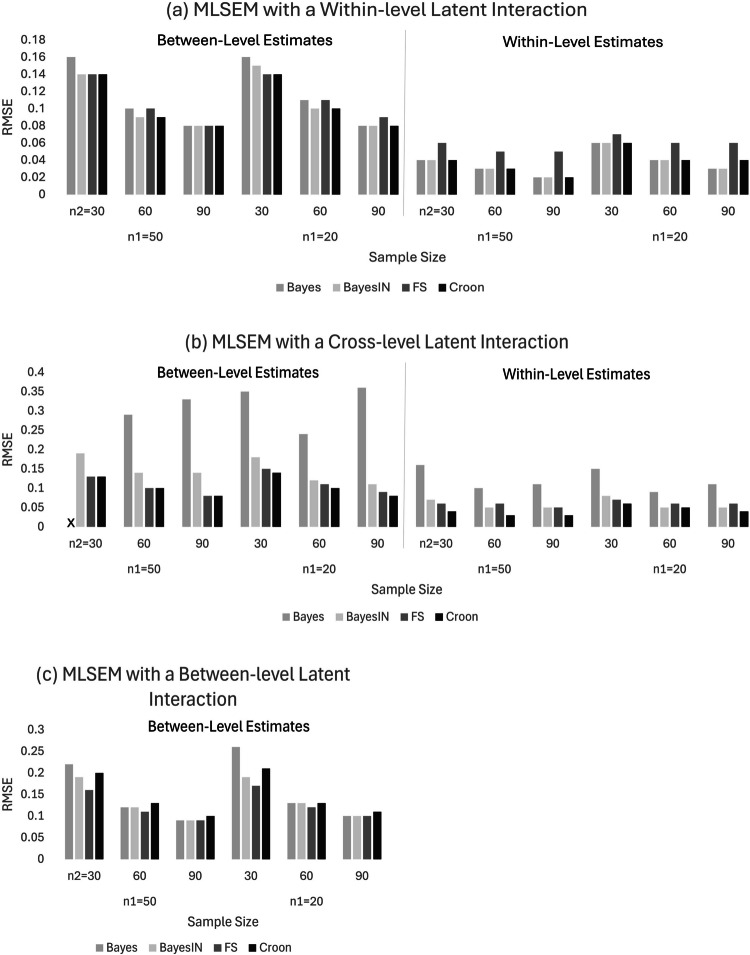
RMSE of estimates from Bayesian and SAM approaches for a multilevel structural equation model with **a** within-level, **b** cross-level, and **c** between-level latent interactions, with ${n}_{1}$=50 and 20 and various ${n}_{2}$=30, 60, and 90

Looking at Table 5 and Fig. 2, we see that the different estimators performed similarly in terms of RMSE for SEMs with between-level and within-level latent interactions, but SAM-Croon performed notably better than other estimators for MLSEMs with cross-level interactions. The RMSE values for estimates of the MLSEM containing the between-level latent interaction produced an example of the trade-offs between bias and efficiency that should be considered during estimator selection. While the SAM-FS approach produced biased estimates, its efficiency led to the lowest RMSE values for this model.

**Table 5:** RMSE for estimates across coefficients at the within and between levels for multilevel structural equation models with within-level, cross-level, and between-level type latent interactions

*Model (level)*	*Sample size*		RMSE all coefficients			
	${n}_{2}$	${n}_{1}$	Bayes	BayesIN	FS	Croon
Within (B)	30	50	0.16	0.14	0.14	0.14
	60		0.10	0.09	0.10	0.09
	90		0.08	0.08	0.08	0.08
	30	20	0.16	0.15	0.14	0.14
	60		0.11	0.10	0.11	0.10
	90		0.08	0.08	0.09	0.08
Within (W)	30	50	0.04	0.04	0.06	0.04
	60		0.03	0.03	0.05	0.03
	90		0.02	0.02	0.05	0.02
	30	20	0.06	0.06	0.07	0.06
	60		0.04	0.04	0.06	0.04
	90		0.03	0.03	0.06	0.04
Cross (B)	30	50	*	0.19	0.13	0.13
	60		0.29	0.14	0.10	0.10
	90		0.33	0.14	0.08	0.08
	30	20	0.35	0.18	0.15	0.14
	60		0.24	0.12	0.11	0.10
	90		0.36	0.11	0.09	0.08
Cross (W)	30	50	0.16	0.07	0.06	0.04
	60		0.10	0.05	0.06	0.03
	90		0.11	0.05	0.05	0.03
	30	20	0.15	0.08	0.07	0.06
	60		0.09	0.05	0.06	0.05
	90		0.11	0.05	0.06	0.04
Between	30	50	0.22	0.19	0.16	0.20
	60		0.12	0.12	0.11	0.13
	90		0.09	0.09	0.09	0.10
	30	20	0.26	0.19	0.17	0.21
	60		0.13	0.13	0.12	0.13
	90		0.10	0.10	0.10	0.11

*Note:* RMSE of estimates are separated by between level (B) and within level (W), with an asterisk (*) indicating that the estimator produced excessive RMSE (e.g., RMSE >1.00)

### Summary

Our first simulation study compared SAM and Bayesian approaches for estimating MLSEMs with three types of latent interactions. Results indicate that SAM-Croon and Bayesian approaches are generally effective in this context, but performance is dependent on the type of latent interaction(s) in the MLSEM and the availability of informative priors for Bayesian approaches. Specifically, Bayesian estimators struggled with cross-level latent interactions while SAM-Croon demonstrated an overall strong performance with models that included this interaction. Even the addition of correctly specified informative priors did not alleviate bias and convergence issues for Bayesian estimation in models with a cross-level interaction. These results support the use of Bayesian approaches for MLSEMs that include a within- or between-level latent interactions if strong and likely accurate priors are available. SAM-Croon is also an appropriate choice in these settings and should be utilized for MLSEMs with possible cross-level latent interactions and in settings where informative priors are unavailable or possibly untrustworthy.

## Partially nested structural equation models

While the additional complexity of fully nested two- or three-level data structures have historically been addressed in the application and development of MLSEMs, more complex data structures are common but have often been ignored (e.g., Mehta & Petscher, 2016). Partially nested data structures are one such common complexity (e.g., Bauer et al., 2008; Lachowicz et al., 2015; Petscher & Schatschneider, 2019; Roberts, 2021; Roberts et al., 2016; Sanders, 2011; Sterba et al., 2014). Partially nested structures arise when, for example, the treatment and control conditions have disparate nesting structures (e.g., multilevel vs. single level). Disregarding partially nested structures (e.g., applying analytic models for single-level or fully nested data) is detrimental to standard error and variance estimate accuracy (e.g., Baldwin et al., 2011; Bauer et al., 2008; Korendijk et al., 2012).

Group-administered interventions and those with a shared facilitator often produce partially nested data, because the treatment condition induces nesting that is not present in a control condition. These types of interventions are prevalent across a broad range of disciplines including education, public health, medicine, psychotherapy, and social work (Lohr et al., 2014; Mehta & Petscher, 2016; Roberts & Walwyn, 2013; Sterba, 2017). For instance, waitlist studies examining the efficacy of therapeutic treatments often introduce a partially nested structure because individuals who participate in the therapy are clustered within therapists while control individuals on a waitlist remain non-clustered or ungrouped.

The prevalence and additional complications of partially nested SEMs make it an ideal example for demonstrating the adaptability of estimation approaches. First, recent advances in SEM provide a theoretical bridge for designs with partially nested structures (e.g., Lachowicz et al., 2015; Sterba et al., 2014). Specifically, literature has extended the MLSEM framework to accommodate analyses with partially nested structures (e.g., Lachowicz et al., 2015; Sterba et al., 2014). However, neither SAM nor Bayesian estimators have been formally extended or evaluated with these partially nested SEMs. Given differences in the operationalization and estimation of main, mediation, and moderation effects in analytic approaches for partially nested data, it is important to extend and investigate estimator performance with these models (Lachowicz et al., 2015; Sterba et al., 2014). Put differently, estimator performance results involving SEMs with latent interactions in simple (fully) nested SEMs cannot be uniformly generalized to partially nested SEMs. With these needs in mind, we describe barriers to extending Bayesian approaches to partially nested SEMs with latent interactions, then present the simple extension of SAM approaches to these settings. Lastly, we investigate the performance of the SAM approaches using a Monte Carlo simulation study.

### Measurement and structural models

**Fig. 3:**
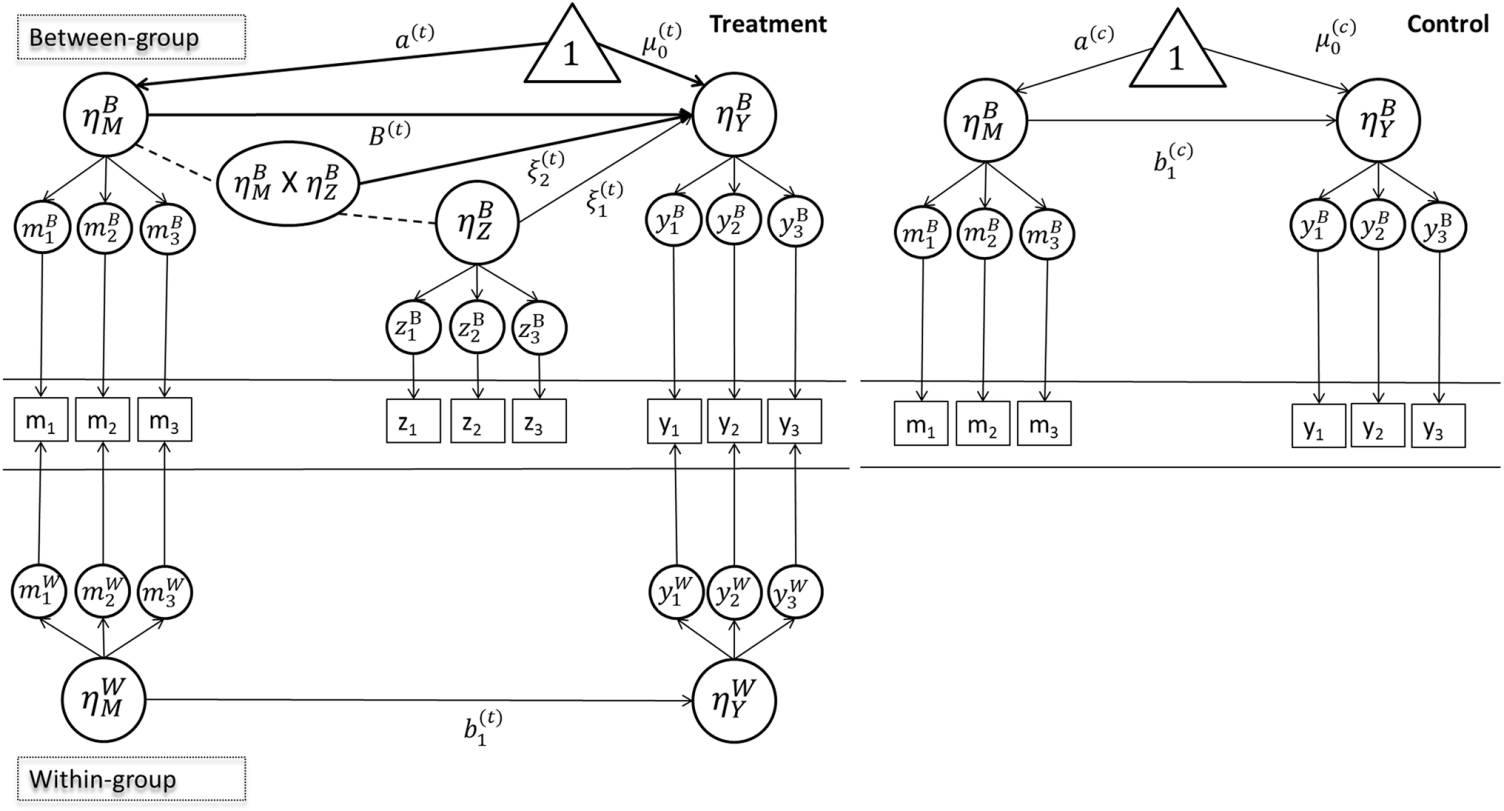
Conceptual representation of a two/one partially nested SEM with moderated mediation

To extend estimators to partially nested structures, we drew on a multiple-arm MLSEM framework for partially nested data (MLSEM-PN; Lachowicz et al., 2015; Sterba et al., 2014). We considered a 2/1 partially nested SEM, where the 2/1 notation reflects a two-level treatment arm and single-level (no grouping or clustering) control arm with latent moderated mediation (see Fig. 3). The previously described factor models (i.e., Eqs. 1 and 2) are still applicable to latent variables in a partially nested SEM. The structural models, however, need to be adapted to reflect the distinct structures in the two study arms. Specifically, the structural model in the treatment arm for the mediator under the MLSEM-PN framework (Lachowicz et al., 2015; Sterba et al., 2014) is shown in Fig. 3 and can be expressed as

15 $$\begin{array}{lc}{\eta}_{{M}_{ij}}^{W(t)}={\pi }_{0}^{(t)}+{\varepsilon}_{{M}_{ij}}^{W(t)}\\ {\eta}_{{M}_{j}}^{B(t)}={a}^{(t)}+{\varepsilon}_{{M}_{j}}^{B(t)}\end{array},$$

where

$$\begin{array}{lc}{\varepsilon}_{{M}_{ij}}^{W(t)}\sim N(0,{\sigma}_{M}^{2(t)})\\ {\varepsilon}_{{M}_{j}}^{B(t)}\sim N(0,{\tau}_{M}^{2(t)})\end{array}$$

Study arm-specific structural models are designated by the superscript (*t*) for the treatment arm and (*c*) for the control arm. Here, we have a level 1 mediator (${M}_{ij}$) in the treatment arm that is decomposed into within- and between-level components (${\eta}_{{M}_{ij}}^{W(t)}$ and ${\eta}_{{M}_{j}}^{B(t)}$, respectively). At the within level, we include the intercept (${\pi}_{0}^{(t)}$) and residual term. Similarly, at the between level we include ${a}^{(t)}$ and the residual term. The residuals parallel those from the fully nested model, with ${\varepsilon}_{{M}_{ij}}^{W(t)}$ the within-level residual and ${\varepsilon}_{{M}_{j}}^{B(t)}$ the between-level residual. The residuals follow a normal distribution, with a mean of 0 and variance of ${\sigma}_{M}^{2(t)}$ and ${\tau}_{M}^{2(t)}$, respectively.

Similarly, the level 1 outcome (${Y}_{ij}$) is decomposed into within- and between-level components (${\eta }_{{Y}_{ij}}^{W(t)}$ and ${\eta }_{{Y}_{j}}^{B(t)}$, respectively), which we model using

16 $$\begin{array}{lc}{\eta}_{{Y}_{ij}}^{W(t)}={\beta}_{0}^{(t)}+{b}_{1}^{(t)}{\eta}_{{M}_{ij}}^{W(t)}+{\varepsilon}_{{Y}_{ij}}^{W(t)} \, \, \, \, \, \, \, \, \, \, \, \, \, \, \, \\ {\eta}_{{Y}_{j}}^{B(t)}={u}_{0}^{(t)}+{B}^{(t)}{\eta}_{{M}_{j}}^{B(t)}+{\xi}_{1}^{(t)}{\eta}_{{Z}_{j}}^{B(t)}+{\xi}_{2}^{(t)}{\eta}_{{M}_{j}}^{B(t)}{\eta}_{{Z}_{j}}^{B(t)}+{\varepsilon}_{{Y}_{j}}^{B(t)}\end{array}$$

where

$$\begin{array}{lc}{\varepsilon}_{{Y}_{ij}}^{W(t)}\sim N(0,{\sigma}_{\left.Y\right\vert}^{2(t)})\\ {\varepsilon}_{{Y}_{j}}^{B(t)}\sim N(0,{\tau }_{\left.Y\right\vert}^{2(t)})\end{array}$$

We have replaced some Greek letters for key variables subsequently used in the estimation of the mediation effect (e.g., *B*, *b*_1_). Specifically, *b*_1_ captures the relationship between the mediator and outcome at the within level in the treatment arm, and *B* captures this relationship at the between level. The $\xi$ coefficients capture other relationships at the between level. Of particular importance is ${\xi}_{2}^{(t)}$, because it is associated with a latent interaction term (${\eta}_{{M}_{j}}^{B(t)}{\eta}_{{Z}_{j}}^{B(t)}$). In this model, there is a hypothesized interaction in the treatment arm between the latent mediator $\left({\eta}_{{M}_{j}}^{B(t)}\right)$ and another between level latent variable $\left({\eta}_{{Z}_{j}}^{B(t)}\right)$. You will see below that this term is excluded from the control arm model by design because the control arm is limited to a single level. Put differently, the between-level latent variable $\left({\eta}_{{Z}_{j}}^{B(t)}\right)$ does not exist in the control arm. For each coefficient associated with a latent predictor, we assume a nonrandom slope. Distributional assumptions for the residual terms parallel those from the mediator model but include conditional variance terms due to the inclusion of covariates at both levels of the outcome model.

The structural model in the control arm is similar to the treatment arm model but must be adapted to reflect the single-level data structure. The structural model for the mediator in the control arm is

17 $$\begin{array}{lc}{\eta}_{{M}_{ij}}^{W(c)}= \, {0} \, \, \, \, \, \, \, \, \, \, \, \, \, \, \\ {\eta}_{{M}_{j}}^{B(c)}={a}^{(c)}+{\varepsilon}_{{M}_{j}}^{B(c)}\end{array}$$

where ${a}^{(c)}$ captures the intercept for the mediator model in the control arm. Note that we have operationalized the structural models for the control arm to handle the single-level data structure by forming clusters (*j*) with one individual per cluster. The within-level expressions (e.g., ${\eta}_{{M}_{ij}}^{W(c)}=0$) are included only to demonstrate this point. The distributional assumptions of the residual are similar to previous models but limited to a single level such that

$${\varepsilon}_{{M}_{j}}^{B(c)}\sim N\left(0,{\sigma}_{M}^{2\left(c\right)}\right),$$

For the outcome model in the control arm we have

18 $$\begin{array}{lc}{\eta}_{{Y}_{ij}}^{W(c)}= \, {0} \, \, \, \, \, \, \, \, \, \, \, \, \, \, \\ {\eta}_{{Y}_{j}}^{B(c)}={\mu}_{0}^{(c)}+{b}_{1}^{(c)}{\eta}_{{M}_{j}}^{B(c)}+{\varepsilon}_{{Y}_{j}}^{B(c)}\end{array}$$

we again utilize *b*_1_ to capture the relationship between the mediator and outcome, but in Eq. 18 this involves participants in the control arm. The distributional assumptions of the residual are similar to the mediator model but again limited to a single level such that

$${\varepsilon}_{{Y}_{j}}^{B(c)}\sim N(0,{\sigma}_{\left.Y\right|}^{2(c)})$$

Two important notes regarding the specification of our moderated mediation model under MLSEM-PN. First, we utilize the study arm as a predictor in our specification. Put differently, we are interested in the effect of the treatment on the outcome as it operates through the mediator (conditional on the moderator). Second, we conceptualize (and analyze) the control arm models as composed of clusters with one individual per cluster, allowing residual variance at the between level only. Under MLSEM-PN, one may estimate residual variance in the control arm at the between level or the within level but not both. Lastly, as with our original structural models for the fully nested SEM, the predictors in the partially nested SEM are multivariate normal, and the outcome is model-implied non-normal.

### Barriers for Bayesian estimation

Currently, the predominant statistical software packages (e.g., R and Mplus) do not naturally accommodate Bayesian or even typical full-information ML estimators for partially nested SEMs with latent interactions. For the measurement and structural models described, a typical long-format data structure (see Sterba et al., 2014) with multiple-group analysis in Mplus works for the SAM approaches, but the integration required for ML to estimate latent interactions is not available and neither is Bayesian estimation. Multiple-group analysis in Mplus with Bayesian estimation is possible using the *knownclass* option under a multilevel mixture model, but this approach does not accommodate partial nesting. We briefly considered a workaround utilizing a modified wide-format data structure with fixed measurement parameters to meet the invariance assumptions typical of fully nested MLSEM and covariances between treatment and control arm variables set to zero. However, this method required long computational runtimes (e.g., > 45 min) and high convergence failure rates, which ultimately made the Bayesian approach infeasible under current constraints.

### Adapting SAM approaches for partially nested SEMs

There are two general areas that require some adaptations to employ SAM approaches with partially nested SEMs. First, our data are structured to reflect the distinct clustered treatment arm and non-clustered control arm and subsequently distinct analytic models. The separation of these data and application of distinct analytic models nullify the need for a treatment indicator. As noted, we include study arm (i.e., treatment and control conditions) as the focal predictor in our mediation path, so we need model specifications that allow estimation of all the parameters required for calculating main, mediation, and moderation effects. Specifically, we utilize measurement models that allow the estimation of latent variable means across study arms in order to estimate differences in the outcome (i.e., main effect) and mediator (i.e., *a* path of the mediation effect). Second, it is necessary to clarify the source of factor scores and other elements from the measurement models used in the correction step of SAM-Croon. Specifically, we utilize study arm-specific factor scores and corrections using the study arm-specific measurement models. The SAM approach disaggregates measurement and structural models for estimation, so any software package capable of accommodating multilevel factor and structural models is suitable for these adaptations.

#### Estimating main, mediation, and moderation effects

Under the MLSEM-PN framework, the difference between outcome latent means across study arms captures the main effect (${\mu}_{0}^{(t)}-{\mu}_{0}^{(c)}$; Lachowicz et al., 2015; Sterba et al., 2014). To calculate this difference using a SAM approach, we estimated the measurement model with both treatment and control participants. Depending on software capabilities, this can be accomplished through a multiple-group approach or a modified wide data structure (Sterba et al., 2014). We employed the latter. This data structure allowed us to set the latent mean of the control condition to zero $\left({\mu}_{0}^{(c)}=0\right)$ and estimate the latent mean of the treatment condition $\left({\mu}_{0}^{(t)}\right)$, which then captures the difference between the two study arms on the outcome. The latent mean of the outcome for the SAM approaches is conditional on the mediator (and covariates). For regression, a general way to obtain the intercept estimate is to take the mean of the outcome and subtract the product of the regression coefficient and mean of the predictor. For SAM-Croon, we used the latent mean of outcome from the full measurement model, the corrected coefficients from the corrected factor score covariance matrix, and the latent means of the predictors from their measurement models to obtain a correct intercept capturing the main effect. SAM-FS follows a similar process but utilizes the uncorrected coefficients from the uncorrected factor score covariance matrix.

As for the mediation effect, we consider an indirect effect in the treatment arm (i.e., total indirect effect). Simple approaches that utilize the product of path coefficients associated with the treatment or focal variable in the mediator model (i.e., *a* path) and mediator in the outcome model (i.e., *b* path) are not possible. Specifically, the typical parameterization of the treatment–mediator relationship (*a* path) is not possible. The structural model does not include a treatment indicator or focal predictor, as treatment and control conditions are separated by design. Under the MLSEM-PN framework, we can still estimate the mediation effect using the product of terms for the *a* path and *b* paths but must adjust the parameters used to estimate these relationships.

First, we can capture the relationship between the treatment and mediator using the difference between the latent means of the mediator in the treatment $\left({a}^{(t)}\right)$ and control $\left({a}^{(c)}\right)$. We use an approach similar to the one utilized for estimating the treatment and outcome relationship (i.e., main effect). Specifically, we estimated the measurement model for the mediator using the full sample (i.e., participants from both treatment and control arms) while setting the latent mean of the mediator for control participants to zero $\left({a}^{(c)}\right)=0$ and freely estimating the parameter for those in the treatment arm. This specification operationalizes $\left({a}^{(t)}\right)$ as the difference between the latent means of the mediator in the treatment arm and control arm (i.e., ${a}^{(t)}-{a}^{(c)}$) and thus captures the relationship of the treatment and mediator (Kelcey et al., 2021; Lachowicz et al., 2015; Sterba et al., 2014).

Second, the mediator–outcome relationship (*b* path) for the total indirect effect is captured by ${B}^{(t)}$ in the outcome model for the treatment arm (see Expression 16; Kelcey et al., 2021; Lachowicz et al., 2015). Our ${B}^{(t)}$ coefficient is an estimate of a treatment condition-specific mediator–outcome relationship (*b* path), and that relationship could be different in the control condition. The difference between the treatment and control condition coefficients (i.e., ${B}^{(t)}-{b}_{1}^{(c)}$) would capture the interaction between the treatment and mediator that modifies the mediator–outcome relationship. For simplicity, we do not consider this interaction and just use ${B}^{(t)}$, as it defines the total indirect effect. The end result is a mediation effect estimated using ${ME={a}^{(t)}B}^{(t)}$. In summary, ${a}^{(t)}$ captures changes in the mediator due to treatment exposure in comparison to the control condition, while ${B}^{(t)}$ captures changes in the outcome due to changes in the mediator in the treatment condition. The product term, ${a}^{(t)}-{B}^{(t)}$ then captures how treatment effects on the mediator ultimately influence the outcome.

Lastly, we describe operationalizing latent interactions in partially nested SEMs. As with the fully nested SEM, we utilized the product of the factor scores for each latent variable that comprised the interaction term to estimate the latent interaction. In our example, we considered a possible interaction that induces a dependency in the mediator–outcome relationship (i.e., *b* path). To be specific, we included a between level-latent variable ${\eta}_{{Z}_{j}}^{B(t)}$ as a possible moderator of the *b* path. As a strictly between-level variable, it is missing from the control arm by design. As with fully nested SEMs, the moderation effect on the “*b* path” can be captured using the coefficient associated with the latent interaction term. In our case, ${\xi}_{2}^{(t)}$ is paired with the latent interaction term ${\eta}_{{M}_{j}}^{B(t)}{\eta}_{{Z}_{j}}^{B(t)}$

#### Study arm-specific corrections

The second adaptation involved the factor score matrices and corrections utilized in the SAM approaches. We estimated study arm-specific measurement models. For both SAM approaches, we used these measurement model results to produce factor score matrices for the between level of the treatment and within level of the treatment and control arm. SAM-FS utilizes the uncorrected factor score matrices to estimate structural model parameters. Measurement model results for the treatment and control arms are used to correct the factor score matrices utilized by the SAM-Croon approach. Using the corresponding factor score matrices, both SAM approaches estimate three structural models: the between-level treatment path model, within-level treatment path model, and a control arm path model. The parameter estimates necessary for calculating the aforementioned effects are found in these path models, specifically the between-level treatment path model.

#### Croon corrections for partially nested SEMs with latent interactions

The process for estimating latent interactions and subsequent corrections for the SAM-Croon approach in partially nested SEMs parallel those for fully nested MLSEM (see the “Croon corrections for multilevel latent interactions” section and Cox et al., 2023). The key for SAM-Croon with partially nested SEMs that include latent interactions is identifying the appropriate corrections using the study arm data structure and type of latent interaction (see Cox & Kelcey, 2021, and Cox et al., 2023). For example, the latent interaction in Eq. 16 is located in the two-level treatment arm, so we apply a multilevel latent interaction correction. Specifically, the two latent variables that form the latent interaction are the mediator located at the within level and the moderator located at the between level. Using Cox et al. (2023), we applied the correction for an interaction involving the between-level component of a variable located at the within level and a variable located solely at the between level (i.e., cross-level latent interaction).

## Partially nested SEM simulation study methods

Our second simulation study generated data based on the above structural models (Fig. 3) and the previously described measurement models (see Table 1 for specific values). We utilized SAM-FS and SAM-Croon to estimate model parameters for 500 datasets per condition and retained all measurement model parameter values. Recall that the superscript (*t*) indicates the treatment arm and (*c*) the control arm (see Eqs. 15-18). In Eq. 16, the coefficient value associated with the moderator variable (Z) was set to ${\xi}_{1}^{(t)}=0.2,$ with the coefficient associated with the latent interaction set at ${\xi}_{2}^{(t)}=0.15$. When generating the latent outcome, we set the intercept in the treatment condition to 0.7 and 0 in the control condition to produce a main effect of 0.7. The mediation effect was set to 0.20 ($ME=0.20$) by generating a treatment–mediator relationship of 0.5 and mediator–outcome relationship of 0.4. Specifically, when generating the latent mediator values in the treatment condition we set the intercept to 0.5 $\left({a}^{(t)}=0.5\right)$ and 0 for the control arm $\left({a}^{(c)}=0.0\right)$, which produces a treatment–mediator (*a* path) relationship of 0.5. We then set ${B}^{(t)}$ from Eq. 16 to 0.4 $\left({B}^{(t)}=0.4\right)$. The ${B}^{(t)}$ coefficient captures the mediator–outcome relationship and, paired with an ${a}^{(t)}$ value of 0.5 $\left({a}^{(t)}{B}^{(t)}\right)$, produces a mediation effect of 0.20.

While they are not necessary for calculation of the mediation effect, other coefficients associated with the mediator were set at ${b}_{1}^{(t)}=0$ and ${b}_{1}^{(c)}=0.4$ in Eqs. 16 and 18, respectively. We retained the decomposition of latent variable variance and indicator variance as ${\sigma}_{\cdot}^{2(t)}=0.8$ and ${\tau}_{\cdot}^{2(t)}=0.2$, with them set at 1.0 in the control arm $\left({\sigma}_{.}^{2(c)}=1.0\right)$. We varied sample size conditions such that ${n}_{2}=30, 40, 50, 80$, and 100, and ${n}_{1}=10, 20, 30$, and 50. In all cases, a balanced design was generated, with ${n}^{(c)}={n}_{2}^{(t)}\times {n}_{1}^{(t)}$. Lastly, we used the same criteria from the first simulation study to assess estimator performance.

## Results

Let us begin with the convergence rate (see Table 6), which we track separately for the treatment and control arms. The simplistic single-level control arm did not have any convergence issues. As for the treatment arm that included the latent interaction, computational effort and runtimes were not an issue. The average time to model convergence was < 1 min in all conditions, while the convergence failure rate never exceeded 5% and was < 3% in all but the smallest sample size condition. It is worth noting that ${n}_{2}^{(t)}$ was an important factor for SAM convergence. The SAM approaches had their highest convergence failure rate when ${n}_{2}^{(t)}=30$ but near 100% convergence in several conditions with larger ${n}_{2}^{(t)}$ values but smaller or similar total sample sizes.

**Table 6:** Convergence failure rate for SAM estimators of a partially nested SEM with latent moderated mediation

			Study arm			
*Sample size*			Treatment		Control	
${n}_{2}^{(t)}$	${n}_{1}^{(t)}$	${n}^{(c)}$	FS	Croon	FS	Croon
30	20	600	4.6%	4.6%	0.0%	0.0%
40	20	800	2.8%	2.8%	0.0%	0.0%
50	10	500	1.8%	2.0%	0.0%	0.0%
50	20	1,000	2.0%	2.0%	0.0%	0.0%
50	30	1,500	1.2%	1.2%	0.0%	0.0%
80	20	1,600	0.0%	0.0%	0.0%	0.0%
80	30	2,400	0.2%	0.2%	0.0%	0.0%
100	10	1,000	0.4%	0.4%	0.0%	0.0%
100	50	5,000	0.0%	0.0%	0.0%	0.0%

*Note:* FS is the SAM uncorrected factor score regression approach (SAM-FS), and Croon is the SAM approach with Croon-based correction of factor score regression (SAM-Croon)

**Fig. 4:**
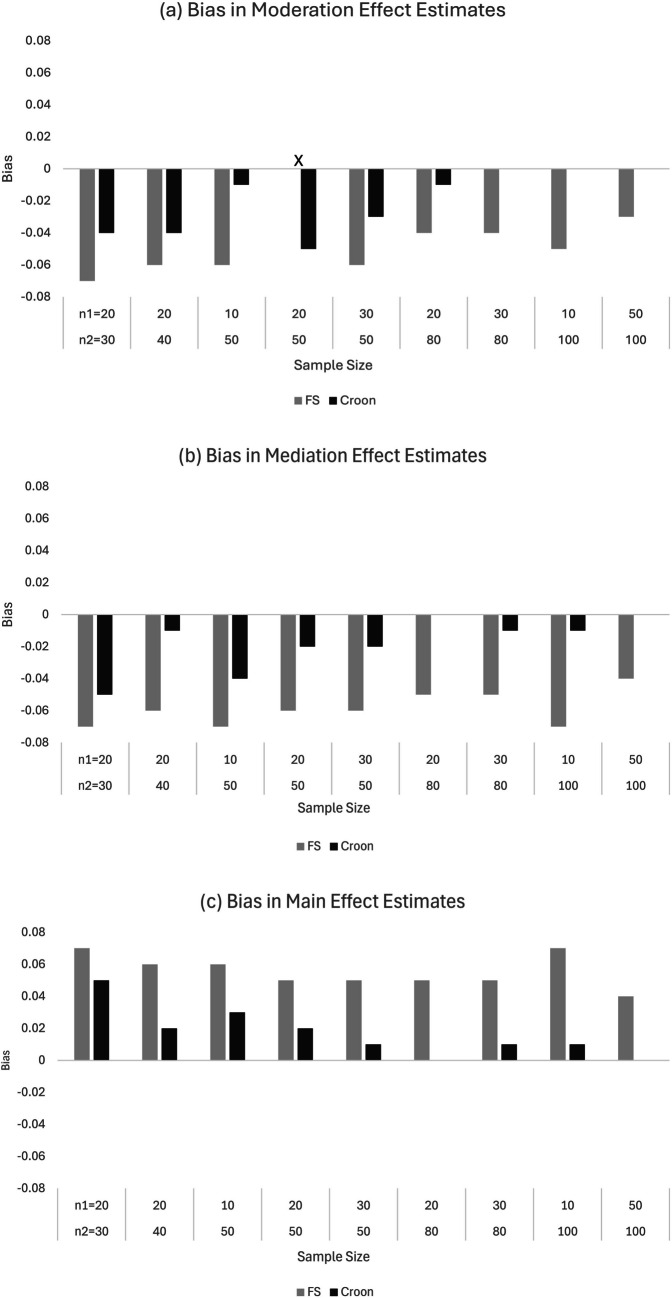
Bias in estimates from SAM approaches of **a** main, **b** mediation, and **c** moderation effects in a two/one partially nested structural equation model with moderated mediation at various ${n}_{1}$ and ${n}_{2}$ sample sizes

When assessing estimator performance in terms of bias, we focused on results at the between level of the treatment arm where effect estimates were located (see Fig. 4 and Table 7). Overall, SAM-Croon avoided bias and produced accurate estimates with smaller sample sizes than SAM-FS, although accurate estimation of the moderation effect was difficult with ${n}_{2}^{(t)}\le 50$ and ${n}_{1}^{(t)}=10 or 20$. When comparing accuracy across effect estimates, we found the moderation effect to be most problematic, followed by the mediation and main effect. Put differently, accurate estimates of the moderation effect required larger sample sizes than the mediation and main effect. The degree of this bias varied by estimator, with SAM-Croon producing smaller discrepancies than SAM-FS. Results for estimator efficiency aligned with expectations (see Table 7). By disregarding measurement error, SAM-FS reduced sampling variability (increased efficiency), while proper accounting for and correcting of measurement error increased sampling variability (decreased efficiency) for SAM-Croon.

**Table 7:** Bias in effect estimates with standard deviation of estimates for the treatment arm of a partially nested SEM with latent moderated mediation

*Est.*	*Sample size*		Estimator			
	${n}_{2}^{(t)}$	${n}_{1}^{(t)}$	FS (*SD*)		Croon (*SD*)	
Mod	30	20	−0.07	(0.41)	−0.04	(0.65)
	40	20	−0.06	(0.32)	−0.04	(0.44)
	50	10	−0.06	(0.42)	−0.01	(0.50)
	50	20	0.13	(4.35)	−0.05	(0.48)
	50	30	−0.06	(0.26)	−0.03	(0.37)
	80	20	−0.04	(0.19)	−0.01	(0.33)
	80	30	−0.04	(0.18)	0	(0.26)
	100	10	−0.05	(0.18)	0	(0.31)
	100	50	−0.03	(0.15)	0	(0.21)
Med	30	20	−0.07	(0.17)	−0.05	(0.27)
	40	20	−0.06	(0.14)	−0.01	(0.21)
	50	10	−0.07	(0.22)	−0.04	(0.24)
	50	20	−0.06	(0.24)	−0.02	(0.21)
	50	30	−0.06	(0.12)	−0.02	(0.17)
	80	20	−0.05	(0.08)	0	(0.12)
	80	30	−0.05	(0.08)	−0.01	(0.12)
	100	10	−0.07	(0.08)	−0.01	(0.14)
	100	50	−0.04	(0.07)	0	(0.10)
Main	30	20	0.07	(0.20)	0.05	(0.30)
	40	20	0.06	(0.17)	0.02	(0.23)
	50	10	0.06	(0.24)	0.03	(0.26)
	50	20	0.05	(0.26)	0.02	(0.23)
	50	30	0.05	(0.14)	0.01	(0.19)
	80	20	0.05	(0.11)	0	(0.14)
	80	30	0.05	(0.11)	0.01	(0.14)
	100	10	0.07	(0.11)	0.01	(0.16)
	100	50	0.04	(0.10)	0	(0.12)

*Note:* Estimates include the moderation effect (Mod), mediation effect (Med), and main effect (Main)

To consider accuracy and efficiency together, we report RMSE values in Table 8 aggregated across levels and study arms. SAM-FS performed well in terms of RMSE at the between level of the treatment arm, mostly due to its efficiency. We again caution that results from SAM-FS are biased as noted above. The model parameters in the second simulation provide an advantageous setting for SAM-FS. The partially nested SEM with moderated mediation has only two path coefficients outside of the between level of the treatment arm, and both are set to 0. The opportunity for SAM-FS to produce (expected) biased results is minimized. Therefore, the strong performance of SAM-FS in terms of RMSE should be viewed with some skepticism.

**Table 8:** RMSE for estimates of a partially nested SEM with latent moderated mediation

*Model*	*Sample size*		RMSE all coefficients	
	${n}_{2}^{(t)}$	${n}_{1}^{(t)}$	FS	Croon
Treat (B)	30	20	0.23	0.34
	40	20	0.19	0.26
	50	10	0.27	0.29
	50	20	0.90	0.26
	50	30	0.16	0.21
	80	20	0.12	0.17
	80	30	0.12	0.15
	100	10	0.13	0.18
	100	50	0.10	0.13
Treat (W)	30	20	0.04	0.05
	40	20	0.04	0.04
	50	10	0.05	0.06
	50	20	0.03	0.04
	50	30	0.03	0.03
	80	20	0.03	0.03
	80	30	0.02	0.03
	100	10	0.03	0.04
	100	50	0.01	0.02
Control	600		0.09	0.06
	800		0.09	0.05
	500		0.09	0.07
	1,000		0.09	0.05
	1,500		0.08	0.04
	1,600		0.08	0.04
	2,400		0.08	0.03
	1,000		0.09	0.05
	5,000		0.08	0.02

*Note:* Model components include the between level of the treatment (Treat (B)), within level of the treatment (Treat (W)), and control arm (Control)

## Discussion

A growing body of research has developed methods to analyze and estimate SEMs with latent interactions. In this study, we investigated the conditions under which competing estimators performed well in both an absolute and relative sense. Specifically, we examined estimator performance with latent interactions located at different levels (i.e., within-, between-, or cross-level interactions) of an MLSEM. The results suggest that estimator performance varies as a function of the location of the interaction (i.e., within-, between-, or cross-level) included in the MLSEM. For estimation of an MLSEM that includes a within-level latent interaction, all estimators except the uncorrected SAM-FS approach are appropriate. We recommend the use of either SAM-Croon or Bayes-IN, as both performed well with this type of model. However, our recommendation of Bayesian approaches does require the availability of accurate and trustworthy priors. For MLSEMs with cross-level interactions, SAM-Croon was the clear preference, as it outperformed the Bayesian estimation with and without informative priors. This condition was particularly difficult for Bayesian approaches under current software constraints. MLSEMs with between-level interactions were the most difficult to accurately estimate with limited samples sizes, but SAM and Bayesian approaches had acceptable overall performance.

The results also suggest that SAM approaches are highly adaptable to different structural models. For example, our study adapted SAM methods for partially nested SEMs that include latent interactions. Specifically, we used SAM approaches to estimate a partially nested SEM with moderated mediation. This serves as an important illustration of the flexibility and adaptability of SAM approaches to increasing model and data complexities. The contribution is substantial due to the unavailability of ML and Bayesian estimation for partially nested SEMs with latent interactions. Simulation results indicated that SAM approaches can achieve appropriate levels of accuracy, efficiency, and feasibility with partially nested SEMs including those with latent interactions. While SAM-FS can serve as a last resort for estimation of between-level effects in very difficult conditions (i.e., very small sample sizes), we do caution against the use of SAM-FS more generally, because it produces systematically biased estimates.

This study is not without limitations. This work serves as a basic comparison of estimator performance for MLSEMs with latent interactions and an initial extension of SAM approaches to partially nested SEMs. The scope of sample size conditions, models, and factors included is limited. This limitation reflects the practical constraints of comparing several estimators in complex SEMs involving latent interactions and a common limitation of all simulation studies. We mitigated these limitations with a supplemental simulation study that varied key factors in a reduced set of conditions (see Supplemental Materials). Nevertheless, we recommend future studies comparing Bayesian and SAM estimation approaches with a more comprehensive collection of conditions varying indicator reliability, variance decomposition of latent variables, and variance decomposition of indicators. These additional results can better inform estimator recommendations, as performance can be more or less robust to these changes. Additionally, future studies should consider a wider variety of SEMs, including those with multiple outcomes and differing measurement models. Lastly, future research should examine estimator performance when model misspecifications are present, as SAM-Croon has demonstrated valuable robustness to certain misspecifications (e.g., Rosseel & Loh, 2024). Conversely, SAM-Croon has shown sensitivity to predictors with non-normal distributions (Rosseel et al., 2025), which warrants further investigation of this and other assumption violations. Lastly, similar to previous extensions of SAM-Croon, our extension does not accommodate random slopes.

## Conclusion

Given the limitations of traditional estimators (e.g., ML) with MLSEMs that include latent interactions, especially when sample sizes are limited, this work study provides important guidance regarding both approaches. Additionally, the extension of SAM approaches to partially nested SEMs with latent interactions represents a compelling advance in the ability of researchers to consider complex theories of action with complex data structures. Specifically, it makes available for partially nested SEMs the compelling strengths of SAM-Croon, which include an ability to function with limited samples sizes, relatively short runtimes, and a variety of model specifications (Cox & Kelcey, 2021, 2023; Cox et al., 2023; Devlieger & Rosseel, 2020; Kelcey et al., 2021; Robitzsch, 2022; Rosseel, 2020; Smid & Rosseel, 2020). Overall, the results improve the analysis of studies with complex theories of action and complex data structures.

## Supplementary Information

Below is the link to the electronic supplementary material.

Supplementary file1 (DOCX 77 KB)

## Data Availability

See Open Practices Statement.
