# Supplementary material for: Comparing Bayesian estimation and structural-after-measurement approaches for structural equation models with latent interactions and complex data structures
Source: Behav Res Methods. 2025 Oct 22;57(11):320. doi: 10.3758/s13428-025-02840-x (PMC12546545; doi:10.3758/s13428-025-02840-x)
Supplement: Supplementary file 1 — Supplementary file1 (DOCX 77 KB) [file 13428_2025_2840_MOESM1_ESM.docx]

# Supplemental Materials

In our full simulation we varied sample size conditions. To ensure these conditions were adequate for an initial investigation of estimator performance we conducted a supplemental simulation that varied factor loadings, residual variance decomposition, and included misspecified priors for BAYES-IN. These factors were varied with MLSEMs that included an A1 within-level and an A2 cross-level latent interaction that matched the primary simulation study. We reduced the factor loadings for $\text{X}_{.}$ and $\text{Z}_{.}$ from 1.0 to 0.66, adjusted the residual variance decomposition of $\sigma^{2}$ and $\tau^{2}$ from $\sigma^{2}=0.8$ and $\tau^{2}=0.2$. to $\sigma^{2}=0.6$ and $\tau^{2}=0.4$. Lastly the misspecified priors for BAYES-IN included priors for the factor loadings of $\text{X}_{.}$ and $\text{Z}_{.}$set at $N(1,1)$ where the true value for the factor loading used to generate data was 0.66. We also set the prior for path coefficients at $N(0.3,1)$ and for the interaction at $N(0.1,1)$ when the true values for used to generate data were 0.4 and 0.2 respectively. Lastly, priors for variance of the latent outcome variable were set at $\sigma^{2}=0.8$ and $\tau^{2}=0.2$ when $\sigma^{2}=0.6$ and $\tau^{2}=0.4$ values were used to generate data.

Results were generally similar between the supplemental simulation and primary simulation (see Tables 1A-5A). We concluded that our primary simulation was sufficient for an initial comparison of Bayesian and SAM approaches for MLSEMs with latent interactions.

## Table 1A

Convergence Failure Rate for Various Estimators of a Multilevel SEM with an A1 Within-level Latent Interaction Under Three Additional Conditions

|  | | Additional Simulation Condition | | | | | | | | | | | | | | | |
| --- | --- | --- | --- | --- | --- | --- | --- | --- | --- | --- | --- | --- | --- | --- | --- | --- | --- |
| Sample Size | | Original | | | | Variance Decomposition | | | | Misspecified Prior | | | | Limited Loading | | | |
| $n_{2}$ | $n_{1}$ | Bayes | BayesIN | FS | Croon | Bayes | BayesIN | FS | Croon | Bayes | BayesIN | FS | Croon | Bayes | BayesIN | FS | Croon |
| 30 | 50 | 0% | 0% | 0% | <1% | 0% | 0% | 0% | 0% | 1% | 0% | 0% | 1% | 0% | 0% | 0% | <1% |
| 60 |  | 0% | 0% | 0% | 0% | 0% | 0% | 0% | 0% | 0% | 0% | 0% | 0% | 0% | 0% | 0% | 0% |
| 90 |  | 0% | 0% | 0% | 0% | 0% | 0% | 0% | 0% | 0% | 0% | 0% | 0% | 0% | 0% | 0% | 0% |
| 30 | 20 | 0% | 0% | 0% | 2% | <1% | 0% | 0% | 0% | 1% | 0% | 0% | 1% | <1% | 0% | 0% | 3% |
| 60 |  | 0% | 0% | 0% | 0% | 0% | 0% | 0% | 0% | 0% | 0% | 0% | 0% | 0% | 0% | 0% | <1% |
| 90 |  | 0% | 0% | 0% | 0% | 0% | 0% | 0% | 0% | 0% | 0% | 0% | 0% | 0% | 0% | 0% | 0% |

Note: Bayesian estimators included naïve priors (Bayes) and informative priors (Bayes-IN), FS is the SAM uncorrected factor score regression approach, and Croon is the SAM approach with Croon based correction of factor score regression. Original indicates the original simulation condition, Variance Decomposition indicates $\sigma^{2}=0.6$ and $\tau^{2}=0.4$. Misspecified Prior indicates the priors for Bayes-IN do not match the values used in the data generation process. The priors for variance are the same from the original study but the actual variance decomposition for latent variables and indicators was 0.6 at the within-level and 0.4 at the between-level. The priors for the coefficients was set at 0.3 while the true value was the original 0.4 and the prior for the latent interaction was set at 0.1 while the true value was the original 0.2. Limited Loading indicates the loadings for all exogenous variables were set at 0.66.

## Table 2A

Convergence Failure Rate for Various Estimators of a Multilevel SEM with an A2 Cross-level Latent Interaction Under Three Additional Conditions

|  | | Additional Simulation Condition | | | | | | | | | | | | | | | |
| --- | --- | --- | --- | --- | --- | --- | --- | --- | --- | --- | --- | --- | --- | --- | --- | --- | --- |
| Sample Size | | Original | | | | Variance Decomposition | | | | Misspecified Prior | | | | Limited Loading | | | |
| $n_{2}$ | $n_{1}$ | Bayes | BayesIN | FS | Croon | Bayes | BayesIN | FS | Croon | Bayes | BayesIN | FS | Croon | Bayes | BayesIN | FS | Croon |
| 30 | 20 | 44% | 5% | 0% | 2% | 48% | 11% | 0% | 0% | 57% | 9% | 0% | 1% | 47% | 7% | 0% | 2% |
| 60 |  | 53% | 7% | 0% | 0% | 55% | 10% | 0% | 0% | 56% | 10% | 0% | 0% | 55% | 12% | 0% | 0% |
| 90 |  | 58% | 27% | 0% | 0% | 56% | 22% | 0% | 0% | 54% | 27% | 0% | 0% | 56% | 26% | 0% | 0% |

Note: Bayesian estimators included naïve priors (Bayes) and informative priors (Bayes-IN), FS is the SAM uncorrected factor score regression approach, and Croon is the SAM approach with Croon based correction of factor score regression. Original indicates the original simulation condition, Variance Decomposition indicates $\sigma^{2}=0.6$ and $\tau^{2}=0.4$. Misspecified Prior indicates the priors for Bayes-IN do not match the values used in the data generation process. The priors for variance are the same from the original study but the actual variance decomposition for latent variables and indicators was 0.6 at the within-level and 0.4 at the between-level. The priors for the coefficients was set at 0.3 while the true value was the original 0.4 and the prior for the latent interaction was set at 0.1 while the true value was the original 0.2. Limited Loading indicates the loadings for all exogenous variables were set at 0.66.

## Table 3A

*Bias in Interaction Coefficient Effect Estimates for Multilevel Structural Equation Models with A1 within-level and A2 cross-level Type Latent Interactions Under Three Additional Conditions*

| *Condition* | *Est.* | *Sample Size* | | Estimator | | | |
| --- | --- | --- | --- | --- | --- | --- | --- |
|  |  | $n_{2}$ | $n_{1}$ | Bayes | BayesIN | FS | Croon |
| Original | A1 | 30 | 50 | 0.00 | 0.00 | -0.03 | -0.01 |
|  |  | 60 |  | 0.00 | 0.00 | -0.03 | -0.01 |
|  |  | 90 |  | 0.00 | 0.00 | -0.04 | -0.01 |
|  |  | 30 | 20 | 0.00 | 0.00 | -0.04 | -0.03 |
|  |  | 60 |  | 0.00 | 0.00 | -0.04 | -0.03 |
|  |  | 90 |  | 0.00 | 0.00 | -0.04 | -0.03 |
|  | A2 | 30 | 20 | -0.16 | 0.02 | -0.05 | -0.02 |
|  |  | 60 |  | -0.03 | 0.02 | -0.04 | -0.01 |
|  |  | 90 |  | -0.13 | 0.02 | -0.04 | -0.01 |
|  |  |  |  |  |  |  |  |
| Variance | A1 | 30 | 50 | 0.00 | 0.00 | -0.03 | -0.01 |
| Decomposition |  | 60 |  | 0.00 | 0.00 | -0.03 | -0.01 |
|  |  | 90 |  | 0.00 | 0.00 | -0.03 | -0.01 |
|  |  | 30 | 20 | 0.00 | 0.00 | -0.04 | -0.03 |
|  |  | 60 |  | 0.00 | 0.00 | -0.04 | -0.03 |
|  |  | 90 |  | 0.00 | 0.00 | -0.04 | -0.03 |
|  | A2 | 30 | 20 | -0.14 | 0.02 | -0.04 | -0.02 |
|  |  | 60 |  | -0.03 | 0.02 | -0.04 | -0.02 |
|  |  | 90 |  | -0.12 | 0.01 | -0.04 | -0.02 |
|  |  |  |  |  |  |  |  |
| Misspecified | A1 | 30 | 50 | 0.00 | 0.00 | -0.03 | -0.01 |
| Prior |  | 60 |  | 0.00 | 0.00 | -0.03 | -0.01 |
|  |  | 90 |  | -0.01 | -0.01 | -0.03 | -0.01 |
|  |  | 30 | 20 | 0.00 | 0.00 | -0.04 | -0.03 |
|  |  | 60 |  | 0.00 | 0.00 | -0.04 | -0.03 |
|  |  | 90 |  | 0.00 | 0.00 | -0.04 | -0.03 |
|  | A2 | 30 | 20 | -0.17 | 0.03 | -0.05 | -0.03 |
|  |  | 60 |  | -0.04 | 0.02 | -0.05 | -0.02 |
|  |  | 90 |  | -0.11 | 0.02 | -0.04 | -0.02 |
|  |  |  |  |  |  |  |  |
| Limited | A1 | 30 | 50 | 0.00 | 0.00 | -0.03 | -0.01 |
| Loading |  | 60 |  | 0.00 | 0.00 | -0.03 | -0.01 |
|  |  | 90 |  | -0.01 | -0.01 | -0.03 | -0.01 |
|  |  | 30 | 20 | 0.00 | 0.00 | -0.04 | -0.03 |
|  |  | 60 |  | 0.00 | 0.00 | -0.04 | -0.03 |
|  |  | 90 |  | 0.00 | 0.00 | -0.04 | -0.03 |
|  | A2 | 30 | 20 | -0.16 | 0.03 | -0.04 | -0.02 |
|  |  | 60 |  | -0.03 | 0.01 | -0.04 | -0.02 |
|  |  | 90 |  | -0.12 | 0.00 | -0.04 | -0.01 |

## Table 4A

*RMSE for Estimators of a Multilevel Structural Equation Models with an A1 within-level Type Latent Interaction Under Three Additional Conditions*

| *Condition* | *Est.* | *Sample Size* | | Estimator | | | |
| --- | --- | --- | --- | --- | --- | --- | --- |
|  |  | $n_{2}$ | $n_{1}$ | Bayes | BayesIN | FS | Croon |
| Original | A1(B) | 30 | 50 | 0.16 | 0.14 | 0.14 | 0.14 |
|  |  | 60 |  | 0.10 | 0.09 | 0.10 | 0.09 |
|  |  | 90 |  | 0.08 | 0.08 | 0.08 | 0.08 |
|  |  | 30 | 20 | 0.16 | 0.15 | 0.14 | 0.14 |
|  |  | 60 |  | 0.11 | 0.10 | 0.11 | 0.10 |
|  |  | 90 |  | 0.08 | 0.08 | 0.09 | 0.08 |
|  | A1(W) | 30 | 50 | 0.04 | 0.04 | 0.06 | 0.04 |
|  |  | 60 |  | 0.03 | 0.03 | 0.05 | 0.03 |
|  |  | 90 |  | 0.02 | 0.02 | 0.05 | 0.02 |
|  |  | 30 | 20 | 0.06 | 0.06 | 0.07 | 0.06 |
|  |  | 60 |  | 0.04 | 0.04 | 0.06 | 0.04 |
|  |  | 90 |  | 0.03 | 0.03 | 0.06 | 0.04 |
|  |  |  |  |  |  |  |  |
| Variance | A1(B) | 30 | 50 | 0.21 | 0.19 | 0.17 | 0.18 |
| Decomposition |  | 60 |  | 0.13 | 0.12 | 0.12 | 0.12 |
|  |  | 90 |  | 0.11 | 0.10 | 0.11 | 0.10 |
|  |  | 30 | 20 | 0.21 | 0.19 | 0.17 | 0.19 |
|  |  | 60 |  | 0.14 | 0.13 | 0.13 | 0.13 |
|  |  | 90 |  | 0.11 | 0.11 | 0.11 | 0.11 |
|  | A1(W) | 30 | 50 | 0.03 | 0.03 | 0.05 | 0.03 |
|  |  | 60 |  | 0.02 | 0.02 | 0.05 | 0.02 |
|  |  | 90 |  | 0.02 | 0.02 | 0.05 | 0.02 |
|  |  | 30 | 20 | 0.05 | 0.05 | 0.07 | 0.05 |
|  |  | 60 |  | 0.03 | 0.03 | 0.06 | 0.04 |
|  |  | 90 |  | 0.03 | 0.03 | 0.05 | 0.03 |
|  |  |  |  |  |  |  |  |
| Misspecified | A1(B) | 30 | 50 | 0.26 | 0.24 | 0.18 | 0.20 |
| Prior |  | 60 |  | 0.15 | 0.16 | 0.13 | 0.14 |
|  |  | 90 |  | 0.11 | 0.11 | 0.11 | 0.11 |
|  |  | 30 | 20 | 0.28 | 0.24 | 0.18 | 0.20 |
|  |  | 60 |  | 0.15 | 0.15 | 0.13 | 0.14 |
|  |  | 90 |  | 0.12 | 0.13 | 0.11 | 0.12 |
|  | A1(W) | 30 | 50 | 0.04 | 0.04 | 0.05 | 0.04 |
|  |  | 60 |  | 0.02 | 0.02 | 0.04 | 0.03 |
|  |  | 90 |  | 0.02 | 0.02 | 0.04 | 0.02 |
|  |  | 30 | 20 | 0.06 | 0.06 | 0.06 | 0.06 |
|  |  | 60 |  | 0.04 | 0.04 | 0.05 | 0.04 |
|  |  | 90 |  | 0.03 | 0.03 | 0.05 | 0.04 |
|  |  |  |  |  |  |  |  |
| Limited | A1(B) | 30 | 50 | 0.16 | 0.15 | 0.15 | 0.16 |
| Loading |  | 60 |  | 0.10 | 0.10 | 0.10 | 0.10 |
|  |  | 90 |  | 0.08 | 0.08 | 0.09 | 0.08 |
|  |  | 30 | 20 | 0.20 | 0.17 | 0.15 | 0.16 |
|  |  | 60 |  | 0.11 | 0.11 | 0.11 | 0.11 |
|  |  | 90 |  | 0.09 | 0.08 | 0.10 | 0.08 |
|  | A1(W) | 30 | 50 | 0.04 | 0.04 | 0.05 | 0.04 |
|  |  | 60 |  | 0.03 | 0.03 | 0.05 | 0.03 |
|  |  | 90 |  | 0.02 | 0.02 | 0.05 | 0.03 |
|  |  | 30 | 20 | 0.07 | 0.07 | 0.07 | 0.07 |
|  |  | 60 |  | 0.05 | 0.05 | 0.06 | 0.05 |
|  |  | 90 |  | 0.04 | 0.04 | 0.05 | 0.04 |

## Table 5A

*RMSE for Estimators of a Multilevel Structural Equation Models with an A2 within-level Type Latent Interaction Under Three Additional Conditions*

| *Condition* | *Est.* | *Sample Size* | | Estimator | | | |
| --- | --- | --- | --- | --- | --- | --- | --- |
|  |  | $n_{2}$ | $n_{1}$ | Bayes | BayesIN | FS | Croon |
| Original | A2(B) | 30 | 20 | 0.35 | 0.18 | 0.15 | 0.14 |
|  |  | 60 |  | 0.24 | 0.12 | 0.11 | 0.10 |
|  |  | 90 |  | 0.36 | 0.11 | 0.09 | 0.08 |
|  | A2(W) | 30 | 20 | 0.15 | 0.08 | 0.07 | 0.06 |
|  |  | 60 |  | 0.09 | 0.05 | 0.06 | 0.05 |
|  |  | 90 |  | 0.11 | 0.05 | 0.06 | 0.04 |
|  |  |  |  |  |  |  |  |
| Variance | A2(B) | 30 | 20 | * | 0.28 | 0.17 | 0.19 |
| Decomposition |  | 60 |  | 0.27 | 0.16 | 0.13 | 0.14 |
|  |  | 90 |  | 0.38 | 0.16 | 0.11 | 0.11 |
|  | A2(W) | 30 | 20 | 0.14 | 0.08 | 0.07 | 0.06 |
|  |  | 60 |  | 0.09 | 0.05 | 0.06 | 0.04 |
|  |  | 90 |  | 0.11 | 0.04 | 0.06 | 0.03 |
|  |  |  |  |  |  |  |  |
| Misspecified | A2(B) | 30 |  | * | 0.33 | 0.19 | 0.22 |
| Prior |  | 60 |  | * | 0.19 | 0.13 | 0.14 |
|  |  | 90 |  | * | 0.16 | 0.11 | 0.12 |
|  | A2(W) | 30 |  | 0.15 | 0.08 | 0.07 | 0.06 |
|  |  | 60 |  | 0.09 | 0.05 | 0.06 | 0.05 |
|  |  | 90 |  | 0.10 | 0.05 | 0.05 | 0.04 |
|  |  |  |  |  |  |  |  |
| Limited | A2(B) | 30 | 20 | * | 0.25 | 0.15 | 0.15 |
| Loading |  | 60 |  | * | 0.15 | 0.11 | 0.10 |
|  |  | 90 |  | 0.35 | 0.16 | 0.10 | 0.09 |
|  | A2(W) | 30 | 20 | 0.16 | 0.09 | 0.08 | 0.07 |
|  |  | 60 |  | 0.10 | 0.06 | 0.06 | 0.05 |
|  |  | 90 |  | 0.11 | 0.06 | 0.05 | 0.04 |
